# Lipid zonation and phospholipid remodeling in nonalcoholic fatty liver disease

**DOI:** 10.1002/hep.28953

**Published:** 2017-02-06

**Authors:** Zoe Hall, Nicholas J. Bond, Tom Ashmore, Francis Sanders, Zsuzsanna Ament, Xinzhu Wang, Andrew J. Murray, Elena Bellafante, Sam Virtue, Antonio Vidal‐Puig, Michael Allison, Susan E. Davies, Albert Koulman, Michele Vacca, Julian L. Griffin

**Affiliations:** ^1^ Department of Biochemistry and Cambridge Systems Biology CentreUniversity of CambridgeCambridgeUnited Kingdom; ^2^ MRC Human Nutrition ResearchCambridgeUnited Kingdom; ^3^ Department of Physiology, Development and NeuroscienceUniversity of CambridgeCambridgeUnited Kingdom; ^4^ Fondazione Mario Negri SudSanta Maria ImbaroChietiItaly; ^5^ Metabolic Research Laboratories, Wellcome Trust‐MRC Institute of Metabolic Science, Addenbrooke's HospitalUniversity of CambridgeCambridgeUnited Kingdom; ^6^ Liver Unit, Department of MedicineCambridge University Hospitals NHS Foundation TrustCambridgeUnited Kingdom; ^7^ Department of HistopathologyCambridge University Hospitals NHS Foundation TrustCambridgeUnited Kingdom

## Abstract

Nonalcoholic fatty liver disease (NAFLD) can progress from simple steatosis (i.e., nonalcoholic fatty liver [NAFL]) to nonalcoholic steatohepatitis (NASH), cirrhosis, and cancer. Currently, the driver for this progression is not fully understood; in particular, it is not known how NAFLD and its early progression affects the distribution of lipids in the liver, producing lipotoxicity and inflammation. In this study, we used dietary and genetic mouse models of NAFL and NASH and translated the results to humans by correlating the spatial distribution of lipids in liver tissue with disease progression using advanced mass spectrometry imaging technology. We identified several lipids with distinct zonal distributions in control and NAFL samples and observed partial to complete loss of lipid zonation in NASH. In addition, we found increased hepatic expression of genes associated with remodeling the phospholipid membrane, release of arachidonic acid (AA) from the membrane, and production of eicosanoid species that promote inflammation and cell injury. The results of our immunohistochemistry analyses suggest that the zonal location of remodeling enzyme LPCAT2 plays a role in the change in spatial distribution for AA‐containing lipids. This results in a cycle of AA‐enrichment in pericentral hepatocytes, membrane release of AA, and generation of proinflammatory eicosanoids and may account for increased oxidative damage in pericentral regions in NASH. *Conclusion*: NAFLD is associated not only with lipid enrichment, but also with zonal changes of specific lipids and their associated metabolic pathways. This may play a role in the heterogeneous development of NAFLD. (Hepatology 2017;65:1165‐1180)

**Abbreviations**
AA: arachidonic acid
DHA: docosahexaenoic acid
EPA: eicosapentaenoic acid
FFA: free fatty acid
GC‐MS: gas chromatography–mass spectrometry
H&E: hematoxylin and eosin
HDoHE: hydroxydocosahexaenoic acid
HEPE: hydroxyeicosapentaenoic acid
HETE: hydroxyeicosatetranoic acid
HFD: high‐fat diet
HODE: hydroxyoctadecadienoic acid
LA: linoleic acid
LC‐MS: liquid chromatography–mass spectrometry
LFD: low‐fat diet
LOX: lipoxygenase
MALDI: matrix‐assisted laser desorption ionization
MCD: methionine/choline‐deficient
MCS: methionine/choline‐sufficient
MSI: mass spectrometry imaging
MUFA: monounsaturated fatty acid
NAFL: nonalcoholic fatty liver
NAFLD: nonalcoholic fatty liver disease
NASH: nonalcoholic steatohepatitis
OPLS‐DA: orthogonal projection to latent structures discriminant analysis
PC: phosphatidylcholine
PCA: principal components analysis
PE: phosphatidylethanolamine
PUFA: polyunsaturated fatty acid
RCD: regular chow diet
SFA: saturated fatty acid
SM: sphingomyelin
TAG: triacylglyceride
WD: Western diet
WT: wild‐type

Nonalcoholic fatty liver disease (NAFLD) is characterized by accumulation of triacylglycerides (TAGs) in the liver (steatosis); however, a subgroup of patients will progress to more serious conditions, including nonalcoholic steatohepatitis (NASH), cirrhosis, hepatocarcinoma, and liver failure.^1^, ^2^, ^3^, ^4^ A “multiple hit” process for NAFLD has been suggested whereby fatty liver is followed by oxidative stress and lipid peroxidation, leading to an inflammation cascade, cell injury, and the development of NASH.^5^ However, the detailed mechanisms underlying disease progression remain poorly understood.

Given that NAFLD is characterized by an imbalance in lipid homeostasis, lipidomics approaches are well placed to interrogate the specific changes associated with this disease.^6^ Previous studies have concentrated on discovering biomarkers to differentiate between nonalcoholic fatty liver (NAFL) and NASH^7^, ^8^, ^9^ and to establish changes to the lipidome with progression of NAFLD.^10^, ^11^ However, many of these previous studies have focused on circulating markers for liver disease and are therefore distal to the actual pathophysiology, or they have relied on tissue extracts that ignore the heterogeneous nature of the disease.

Different zones exist in the liver lobules according to proximity to the portal triad, the main supplier of nutrient and oxygen‐rich blood to the liver. In addition to a gradient of nutrients and oxygen, zonation of glucose and fatty acid metabolism has been observed.^12^ Whereas hepatocytes closest to the portal vein (periportal or zone 1) are more involved in gluconeogenesis and β‐oxidation of fatty acids, glycolysis and lipogenesis occur at a higher rate in hepatocytes closest to the central vein (pericentral or zone 3).^12^ Furthermore, steatosis in adult NAFL and oxidative damage to cells in NASH are chiefly localized to pericentral regions.^1^ As a consequence, lipids are distributed differentially within the liver lobule; however, little is known about how the development and progression of NAFLD affects lipid zonation and metabolism. Proof‐of‐principle studies have shown that mass spectrometry imaging (MSI), a transformative technology enabling the *in situ* analysis of tissue molecular composition,^13^, ^14^, ^15^ can detect the differential distribution of lipids in both healthy and steatotic liver tissue.^16^, ^17^, ^18^

In this study, we analyzed dietary and genetic models of NAFL and NASH in mice to complement a set of human liver biopsies. Using matrix‐assisted laser desorption ionization (MALDI) MSI coupled with lipidomics techniques and bioinformatics tools, we monitored subtle changes in the distribution and abundance of lipids as a function of their location within the liver lobule. We found several lipids with distinct distributions in control and NAFL liver, along with an increase in proinflammatory arachidonic acid (AA)‐derived lipoxygenase (LOX) metabolites. Progression to NASH, however, led to increasing loss of lipid zonation.

We further found increased hepatic messenger RNA (mRNA) levels in both mouse and human NAFLD samples for genes associated with membrane remodeling (*LPCAT2, cPLA2*) and eicosanoid production (*ALOX15*). Additionally, immunostaining revealed that the LPCAT2 protein, which is responsible for incorporation of AA into the membrane, had a distinct pericentral distribution in human liver. Up‐regulation of LPCAT2 in NAFLD may therefore be a potential driving force behind enrichment of AA in pericentral hepatocytes, enabling the phospholipid membrane to serve as a substrate pool for free AA and its eicosanoid metabolites. This study is the first to suggest the potential importance of the physical location of intact lipids and their associated metabolic pathways in the pathology of NAFLD.

## Materials and Methods

### MOUSE SAMPLES

#### High‐Fat Diet

Five‐week old male *ob/ob* (n = 5) and wild‐type (WT, C57BL/6J strain, n = 5) mice were maintained on a high‐fat diet (HFD) or regular chow diet (RCD) for 12 weeks. The caloric content of the HFD was 55% fat, 29% protein, and 16% carbohydrate (diet code 829197; Special Diet Services, Witham, UK). The caloric content of the RCD was 11.5% fat, 26.9% protein, and 61.6% carbohydrate (RM1; Special Diet Services).

#### Western Diet

Eight‐week old male WT (C57BL/6J strain) mice were maintained on a Western diet (WD) (n = 5/time point; adjusted calories diet code TD.88137; caloric content = 42.0% fat, 15.2% protein, 42.7% carbohydrate, 0.2% cholesterol; Harlan Laboratories, Madison, WI) or low‐fat control diet (n = 5/time point; TD 08485; caloric content: 13% fat, 19.2% protein, 67.9 % carbohydrate; Harlan Laboratories) for 12 or 32 weeks.

#### Methionine/Choline‐Deficient Diet

Eight‐week old male WT mice (FVB/N strain) were fed a methionine/choline‐deficient (MCD) HFD (n = 5; D12451; Research Diets, New Brunswick, NJ) or a methionine/choline‐sufficient (MCS) control diet (n = 5) for 8 weeks as described previously.^19^

#### Liver Tissue and Blood Serum Collection

Mice were euthanized, and the liver tissue was rapidly dissected and snap‐frozen. Liver enzyme activity assays were performed on collected serum. All animal protocols were approved by the UK Home Office and the University of Cambridge Animal Welfare and Ethical Review Board and were performed by a personal licence holder.

### HUMAN SAMPLES

Twenty‐three human samples were obtained from the Human Research Tissue Bank at Addenbrooke's Hospital, Cambridge, United Kingdom (Cambridgeshire 2 Research Ethics Committee, NRES 11/EE/0011). Samples were scored contemporaneously by an experienced histopathologist for steatosis (0‐3), ballooning (0‐2), inflammation (0‐2), and fibrosis (0‐4) and were diagnosed according to the SAF system.^20^ Samples were thus classified as normal (n = 2), NAFL (n = 11), borderline minimal NASH (n = 4), moderate NASH (n = 5) and cirrhosis (n = 1). All samples were analyzed, but for the purposes of statistics, comparisons were made between two groups: NAFL (n = 11) and borderline NASH/NASH (n = 9), referred to hereafter as NASH.

### MALDI‐MSI

Mouse and human liver samples were embedded in Tissue‐Tek OCT, and 10‐μm frozen sections were placed on glass microscope slides and prepared using a cryostat. Adjacent sections were stained with hematoxylin and eosin (H&E) or Masson's trichrome. Tissue sections were washed with 50 mM potassium nitrate (5 s) before matrix application to form exclusively potassiated adducts (Supporting Fig. S1A) to reduce spectral complexity (a combination of K^+^, Na^+^, and H^+^ adducts are normally detected with MALDI). Matrix solutions (10 mg/mL) of 2,5‐dihydroxybenzoic acid (Sigma‐Aldrich, St. Louis, MO) were prepared in 85:15 methanol/water (v/v) and administered to the tissue surface using a nebulized sprayer (Suncollect MALDI spotter; KR Analytical, Cheshire, UK). Imaging experiments were performed for three biological replicates per group (mouse) and all human samples using a MALDI LTQ Orbitrap XL (Thermo Fisher Scientific, Hemel Hempstead, UK) at 50‐μm increments across the tissue. Spectra were acquired in positive ion mode from 250‐1000 *m/z* at 60,000 resolution. Lipid identity was performed by accurate mass using the LipidMaps database.^21^ The predominant fatty acid composition was confirmed where possible by tandem MS using collision‐induced dissociation. To improve fragmentation, lithium adducts with more efficient fragmentation^22^ were formed by a 5 s washing step in lithium nitrate (50 mM) before deposition of matrix, and the corresponding lithiated ions were subjected to fragmentation (Supporting Fig. S1B).

### LIPIDOMICS

Lipids were extracted using an adaptation of the Folch method.^23^ Briefly, 400 μL of deionized water was added to 30 mg of liver tissue and homogenized using a TissueLyser (Qiagen Ltd., Manchester, UK). Chloroform/methanol (2:1, 1 mL) was added and the samples vortexed and centrifuged (12,000*g*, 10 minutes). The extraction was performed twice, and the resulting organic layers were combined, dried under nitrogen, and reconstituted in chloroform/methanol (2:1, 300 µL). Before analysis, samples (20 μL) were diluted in isopropanol/acetonitrile/water (2:1:1, 980 µL).

Samples were analyzed using liquid chromatography–mass spectrometry (LC‐MS) using an Accela Autosampler (Thermo Fisher Scientific) coupled to a LTQ Orbitrap Elite (Thermo Fisher Scientific). Five microliters of sample were injected onto an Acuity C18 BEH column (Waters Ltd., Warrington, UK; 50 × 2.1 mm, 1.7 µm) maintained at 55 °C. Mobile phase A was 10 mM ammonium formate in acetonitrile/water (60:40) and mobile phase B was 10 mM ammonium formate in isopropanol/acetonitrile (90:10). The flow rate was 0.5 mL/min; the mobile phase gradient is detailed in Supporting Table S1. A heated electrospray ionization source was maintained at 375 °C, the desolvation temperature was 380 °C, and the desolvation gas flow was set at 40 arbitrary units. Spectra were acquired in positive and negative ion mode in the range of 100‐2000 *m/z*.

### GAS CHROMATOGRAPHY–MASS SPECTROMETRY

Liver tissue or pellets of feed were extracted as described above. Total fatty acids in the dried lipid extract (200 µL) were derivatized using methanolic boron trifluoride (14%, 125 µL). Chloroform/methanol (1:1, 100 µL) was added, and the samples were heated to 80**°**C for 90 minutes. After cooling, deionized water (300 μL) and hexane (600 μL) were added to each sample. The upper organic fractions were separated, dried under nitrogen, and reconstituted in hexane (200 μL) for analysis.

A 7683 series autosampler (Agilent Technologies, Santa Clara, CA) coupled to a 7683B Injector (Agilent Technologies) was used to inject 1 μL of sample onto an HP88 GC column (Agilent Technologies) (88% cyanopropyl aryl‐polysiloxane, 0.17 μm thickness, 320 μm diameter, 50 m length). The injector temperature was 250 °C using a split ratio of 10:1, and the flow rate of helium was 12 mL/min. An initial temperature of 100**°**C was held for 1 minute, followed by a temperature ramp of 10**°**C/min to 300**°**C, maintained for 2 minutes. Data were acquired after a solvent delay of 4 minutes (60‐400 *m/z*).

### EICOSANOID ASSAY

Eicosanoids were extracted from homogenised tissue (50 mg) as described previously.^24^ Samples were analyzed using LC‐MS/MS on a 5500 QTRAP (ABSciex UK, Warrington, UK) coupled to an ACQUITY system (Waters Ltd.). Samples were injected (10 μL) onto a Kinetex 2.6 µm XB‐C18 100 Å column (100 × 2.1 mm, Phenomenex, Macclesfield, UK) at 30 °C. Mobile phase A consisted of 0.1% acetic acid in water, whereas mobile phase B was 0.1% acetic acid in 80:20 acetonitrile/methanol (0.5 mL/min, gradient in Supporting Table S2). The electrospray source was operated in negative ion mode (4.5 kV and 650 °C). Mass transitions of 51 analytes and three isotopically labeled standards (Supporting Table S3) were acquired. Peaks were integrated within Analyst version 1.6 and normalized against wet tissue weight and isotopically labeled internal standard; quantification was performed using available standards (Cayman Chemical Company).

### TRANSCRIPT QUANTIFICATION

Total RNA was purified from frozen human and mouse liver using an RNeasy Mini Kit (Qiagen). Briefly, approximately 20 mg of tissue samples were lysed and homogenized in Trizol (1 mL) using a TissueLyzer (Qiagen). The samples were centrifuged at 12,000*g* for 15 minutes after the addition of chloroform (200 µL) and the RNA‐containing aqueous phase combined with 1 volume of 70% ethanol. Samples were loaded on spin columns and the procedure was performed according to the manufacturer's guidelines. Purified RNA concentration was quantified (260 nm) using a NanoDrop 100 (Thermo Fisher Scientific). Genomic DNA contamination was eliminated using RT^2^ First Strand Kit (Qiagen) and complimentary DNA was produced using an RT^2^ First Strand Kit (Qiagen). Relative abundance of transcripts of interest was assessed using quantitative polymerase chain reaction in RT^2^ SYBRgreen Mastermix (Qiagen) with a StepOnePlus detection system (Applied Biosciences, Warrington, UK). RT^2^ primer assays for mouse *Rn18s* (endogenous control); c*Pla2, Lpcat2, Alox5, Alox12, Alox15, Tnf‐α, Tgb‐β1*; and human *ACTB* (endogenous control), c*PLA2, LPCAT2*, and *ALOX15* were obtained from Qiagen. Thermocycler (PTC‐200, MJ Research) parameters were as follows: incubation, 95 °C for 10 minutes; elongation, 95 °C for 15 seconds; and cooling, 60 °C for 1 minute. Elongation and cooling were performed in 40 cycles. Expression levels were normalized to endogenous controls using the ΔΔC_T_ method.

### IMMUNOHISTOCHEMISTRY

Immunostaining was performed on 10‐μm frozen sections fixed in cold acetone using the Rabbit VECTASTAIN ELITE ABC horseradish peroxidase kit (Vector Laboratories Ltd, Peterborough, UK) following the manufacturer's protocol. Counterstaining was performed using hematoxylin (Sigma‐Aldrich). Primary antibodies were anti‐LPCAT2 (Atlas Antibodies, Stockholm, Sweden; HPA007891; 1:200 [mouse], 1:500 [human]) and anti‐CD68 (Abcam, Cambridge, UK; ab955; 1:250).

### DATA PROCESSING

#### Lipidomics

Data were converted to mzML format for subsequent data processing, and features were picked using an in‐house R script. Principal components analysis (PCA) and orthogonal projection to latent structures discriminant analysis (OPLS‐DA) models^25^, ^26^ were constructed using SIMCA 14 (Umetrics, Sweden), following normalization to the total ion count and Pareto scaling.^27^

#### MALDI‐MSI

Each MALDI‐MSI experiment comprised multidimensional data whereby every pixel (*x, y* coordinate) was associated with a mass spectrum (*m/z*, intensities). Data from MALDI‐MSI were converted to imzML format for processing.^28^ Using an in‐house R script, ions were retained above a threshold intensity and the *m/z* ratio of ions detected were summed across integral regions of 10 ppm to allow for alterations in *m/z* mass accuracy across the experiment (“binning”). Ion intensities were normalized to total ion count, mean centered, and Pareto scaled before production of single ion images or multivariate statistical analysis (PCA). During PCA, pixels were considered analogous to observations, and *m/z* values were considered analogous to variables. Principal components loadings were normalized according to their contribution to the overall variation before computing averages from different experiments. Multi‐ion image overlays were produced using ImageQuest software (Thermo Fisher Scientific).

#### Univariate Statistics

Unless stated otherwise, data are reported as the mean ± standard error of the mean. Statistical significance was evaluated using analysis of variance with Tukey's *post hoc* test.

## Results

### HEPATIC LIPID SIGNATURES CHANGE WITH NAFL

**Figure 1:**
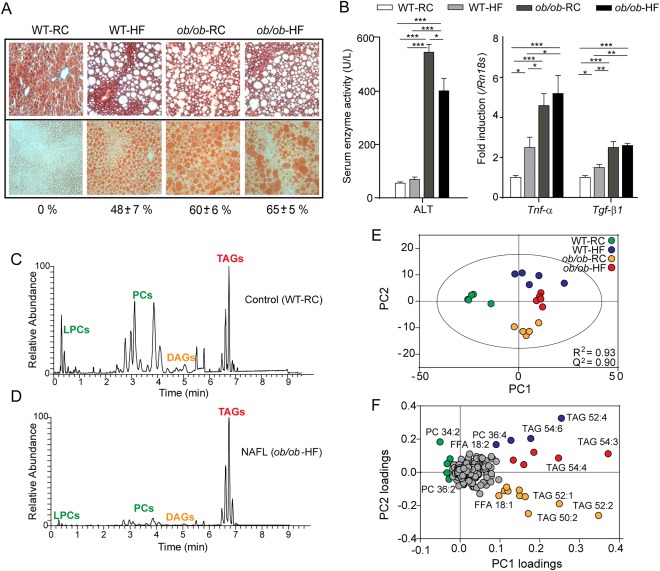
Hepatic lipid signatures in murine NAFL. (A) Increasing steatosis (WT‐RCD < < WT‐HFD < *ob/ob*‐RCD ≈ *ob/ob*‐HFD) was assessed using Masson's trichrome staining (top) and quantified (%) using oil red O lipid stain (bottom). Original magnification: × 200. (B) Serum alanine aminotransferase levels were elevated in *ob/ob*‐RCD and *ob/ob*‐HFD groups, and a stepwise increase in hepatic mRNA levels for *Tnf‐α* and *Tgf‐β1* was observed in WT‐HFD, *ob/ob*‐RCD, and *ob/ob*‐HFD groups. *P < 0.05. **P < 0.01. ***P < 0.001. (C, D) Representative LC‐MS chromatograms are shown for control (WT‐RCD) (C) and NAFL (*ob/ob*‐HFD) mouse liver (D). (E) Lipids identified by LC‐MS were used to construct principal components models, which clearly distinguish the four groups based on their lipid signatures. (F) The associated loadings plot shows the most important lipids for differentiating groups.

We induced NAFL in mice using dietary and genetic models: WT and *ob/ob* mice were maintained on a HFD or RCD. WT (C57BL/6J strain) mice are susceptible to diet‐induced obesity and develop NAFL when fed a HFD,^29^ whereas *ob/ob* mice lack the ability to produce leptin, presenting a phenotype of obesity and insulin resistance, and spontaneously develop steatosis.^29^, ^30^ Body weights for the four groups were measured after 12 weeks and were found to increase in the following order: WT‐RCD < WT‐HFD < < *ob/ob*‐RCD < *ob/ob*‐HFD.^31^ NAFL was induced in the WT‐HFD and *ob/ob* mice and was characterized by zone 3 macrovesicular steatosis, with the amount of steatosis greatest in the *ob/ob*‐RCD and *ob/ob*‐HFD groups (Fig. 1A). Early signs of inflammation and cell injury were observed (Fig. 1B), including increased liver enzymes and expression of proinflammatory cytokine *Tnf‐α* and pro‐fibrotic factor *Tgf‐β1*.

To determine whether the lipid signatures in liver tissue would enable the distinction of the aforementioned groups, we performed lipidomics experiments. Lipids were extracted from homogenized tissue and analyzed using LC‐MS. Overall, in positive and negative ion modes, over 800 lipids were identified from 11 classes, including TAGs, diacylglycerides, phosphatidylcholines (PCs), phosphatidylethanolamines, phosphatidylinositols, phosphatidylglycerols, phosphatidylserines, sphingomyelins (SMs), cholesteryl esters, and free fatty acids (FFAs). As expected, the most striking observation was the relative increase in TAGs compared with PCs in NAFL liver compared with control (Fig. 1C,D). Identified lipids were used to construct a PCA model, which successfully discriminated the four sample groups (R^2^ = 0.93, Q^2^ = 0.90; Fig. 1E). The first principal component related primarily to the disease status (NAFL or normal), whereas the second principal component differentiated the diets. The corresponding loadings plot (Fig. 1F) revealed the lipid species most influential in distinguishing groups. These included a relative increase in PCs and saturated fatty acids (SFAs) for WT‐RCD control liver tissue and a relative increase in TAGs and monounsaturated fatty acids (MUFAs), particularly FFA(18:1), for the NAFL groups. The HFD groups had a greater proportion of polyunsaturated fatty acids (PUFAs) and TAGs with longer chain length and greater unsaturation. In contrast, the *ob/ob*‐RCD group had the highest FFA(18:1) content of all four groups as well as shorter chain TAGs.

Next, we performed gas chromatography–mass spectrometry (GC‐MS) to determine the total fatty acid profiles of liver tissue from the different groups and to compare these with the profiles from the HFD and RCD feed (Supporting Fig. S2, Supporting Table S4). Interestingly, the MUFA/SFA ratio was similar in the HFD and RCD feed but increased up to 4‐fold in *ob/ob*‐RCD mice compared with WT‐RCD controls. This suggests that the increased hepatic MUFA is due to an increase in production through *de novo* lipogenesis^4^ and SCD1 activity. We calculated a proxy for increased SCD1 activity^32^ and found this to be increased in *ob/ob* compared with WT mice, with the greatest increase observed in *ob/ob*‐RCD mice (Supporting Table S4). There was a high proportion of PUFAs in both diets, and consequently the HFD‐fed and WT‐RCD mice had similarly high PUFA content. The *ob/ob*‐RCD mice, however, had significantly lower PUFAs compared with diet composition, presumably as a consequence of increased *de novo* lipogenesis and MUFA production. In conclusion, we have determined differences in both fatty acid content and intact lipid species in liver tissue that are not attributable to diet alone and are likely to arise from NAFLD or the metabolic context from which NAFLD arises.

### NAFL IS ASSOCIATED WITH CHANGES TO LIPID ZONATION

**Figure 2:**
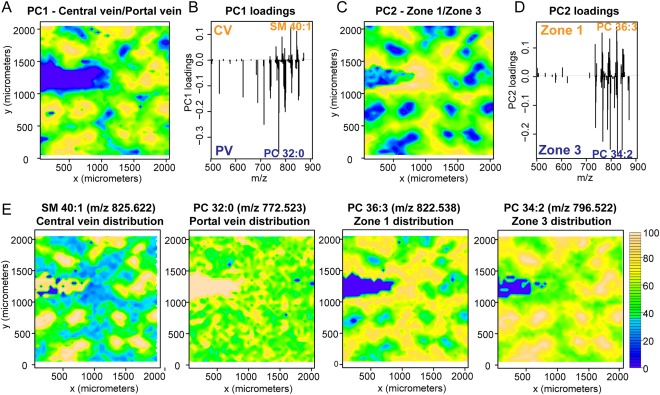
PCA of imaging data reveal liver histology. PCA was employed to evaluate the spatial distribution of lipids, illustrated here for control (WT‐RCD) mouse liver tissue. The PCA scores for the first two principal components were plotted against *x* and *y* coordinates (yellow and blue colors indicate the most positive and negative scores, respectively). (A‐D) Contour plots correspond to regional differences in central vein (CV)/portal vein (PV) (A) and zone 1/zone 3 (C). The corresponding loading plots (B, D) represent which lipids (*m/z*) are most important for differentiating regions. (E) Two‐dimensional single ion distributions for SM(40:1) [M+K^+^], PC(32:0) [M+K^+^], PC(36:3) [M+K^+^], and PC(34:2) [M+K^+^] show a central vein, portal vein, zone 1, or zone 3 distribution, respectively. The highest intensities are shown in yellow.

Next we sought to address whether lipids were differentially distributed in WT‐RCD control liver and how such zonal patterns were affected with NAFL. Using MALDI‐MSI, lipids were directly ablated from the surface of tissue, retaining spatial information that could be linked back to histology. Typically, MALDI spectra of liver tissue (Supporting Fig. S1A) were composed primarily of potassiated PCs and their corresponding fragment ions (Supporting Tables S5, S6). PCA was employed to comprehensively evaluate the spatial distribution of lipids in an unsupervised manner. PCA scores were visualized as a contour plot for each principal component. The first two principal components typically corresponded to histological features of interest with further components, representing successively less variation, attributed to artifacts of matrix deposition and tissue/matrix borders (Figure 2A, C). The loadings plot associated with each principal component represents the lipid species most important for discriminating regions (Figure 2B, C). For instance, SM(40:1) (*m/*z 825.622; [M+K^+^]) and PC(32:0) (*m/z* 772.523; [M+K^+^]) were predominantly located in the central vein or portal vein, respectively Fig. 2E. This was reflected in the scores and loadings plot for the first principal component. Zones 1 and 3 could be distinguished based on their lipid profiles and subsequent PCA scores in the second principal component Fig. 2C, D. In particular, PC(36:3) (m/z 822.538; [M+K^+^]) showed a strong zone 1 distribution, whereas PC(34:2) (m/z 796.522; [M+K^+^]) was predominantly located in zone 3 (Fig. 2E). Method reproducibility was assessed by comparing across serial sections from the same sample (Supporting Fig. S3).

**Figure 3:**
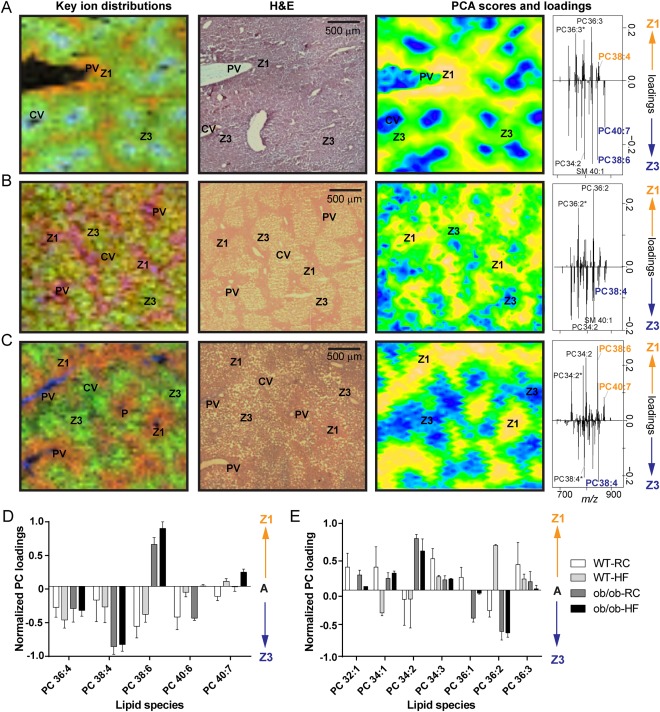
Lipid zonation in murine NAFL. Sections of mouse liver tissue were probed by MALDI‐MSI or stained with H&E. Two‐dimensional distributions for key ions (left) and PCA scores plots (right) closely recapitulate liver histology (middle). (A‐C) Key ion distributions show PC(36:3) [M+K^+^] (red), PC(34:2) [M+K^+^] (green), and SM(40:1) [M+K^+^] (blue) for WT‐RCD control mice (A); PC(36:2) [M+K^+^] (red), PC(34:2) [M+K^+^] (green), and PC(32:0) [M+K^+^] (blue) for WT‐HFD mice (B); and PC(38:6) [M+K^+^] (red), PC(38:4) [M+K^+^] (green), and PC(32:0) [M+K^+^] (blue) for *ob/ob*‐HFD mice (C). Positive and negative PCA scores are associated with zones 1 and 3, respectively (yellow and blue colours indicate the most positive and negative scores, respectively). PCA loadings plots (far right) show the lipids of greatest importance for differentiating zones. Histological features of interest are labeled (CV, central vein; PV, portal vein; Z1, zone 1; Z3, zone 3); *m/z* peaks labeled with asterisks are fragment ions. (D, E) Zonation for different lipid species was quantified across biological replicates using PC loadings scores.

We went on to compare the WT‐RCD liver tissue (Fig. 3A) with tissue from NAFL groups with different extents of steatosis and inflammation (Fig. 3B,C). Using the PCA scores and loadings plots and cross‐referencing to adjacent H&E‐stained sections (Fig. 3), we identified lipids with strong and reproducible zone 1 or zone 3 distributions (Fig. 3D,E). Whereas PC(32:0) and SM(40:1) were typically the major lipids observed in portal vein and central vein, respectively, for all groups, changes were observed in the lipids located in zones 1 and 3 (Fig. 3D,E). For instance, PC(34:2) was distributed mainly in zone 3 in WT‐RCD and WT‐HFD mice (Fig. 3A) but in zone 1 in *ob/ob*‐HFD (Fig. 3C,E). Of particular interest were AA‐containing PC(38:4) (*m/z* 848.553; [M+K^+^]; fatty acid composition 18:0/20:4), which became more strongly localized in zone 3 for *ob/ob* mice, and docosahexaenoic acid (DHA)‐containing PC(38:6) (*m/z* 844.522; [M+K^+^]; 16:0/22:6) which shifted from zone 1 in WT mice to zone 3 in *ob/ob* mice (Fig. 3D; Supporting Fig. S4). This was mirrored to a certain extent by PC(40:7) (16:1/22:6) and PC(40:6) (16:0/22:6) (Fig. 3D).

### NASH LEADS TO FURTHER INCREASE IN MONOUNSATURATED FAT AND LOSS OF ZONATION

Having assessed the changes to lipid abundances and distributions in NAFL mice, we similarly assessed mouse models of NASH. Initially, we used the well‐established MCD diet in mice to model NASH. We first assessed the hepatic lipid profile of MCD mice compared with their MCS controls by LC‐MS (Supporting Fig. S5). We observed increased FFA(22:6) and long‐chain TAGs containing 22:6, whereas MUFAs were decreased or unchanged. Coupled with the knowledge that the MCD challenge induces weight loss, we interpret these results as a possible consequence of adipose tissue lipolysis.^32^, ^33^ We therefore opted for a WD to model NASH because this can recapitulate the metabolic features of human NASH while inducing obesity in mice.^34^, ^35^, ^36^

**Figure 4:**
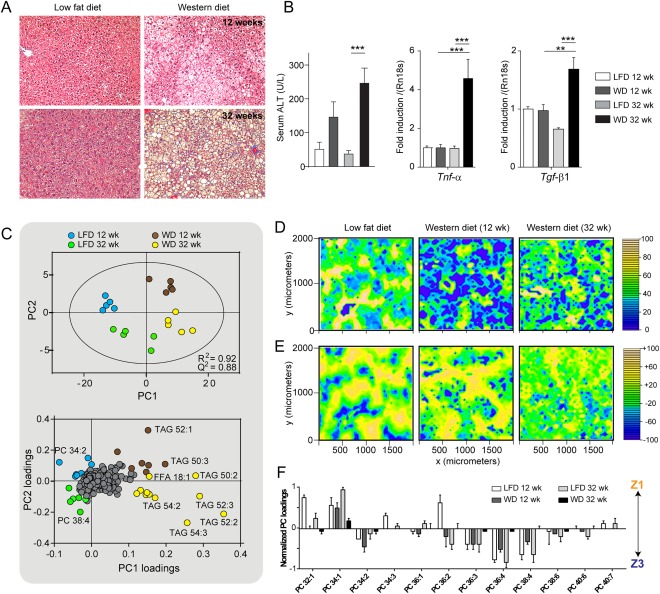
Lipid changes in a mouse model of NASH. (A) Masson's trichrome staining (magnification × 100) revealed that liver tissue from WT mice on a WD for 12 weeks developed steatosis, and after 32 weeks developed NASH with fibrosis (blue stain). (B) Serum alanine aminotransferase and hepatic transcripts *Tnf‐α* and *Tgf‐β1* were elevated in 32‐week WD mice compared with LFD controls and 12‐week WD mice. **P < 0.01. ***P < 0.001. (C) PCA and corresponding loadings plot for distinguishing the groups based on their hepatic intact lipid profiles determined by LC‐MS. (D) Spatial distribution of SM(40:1) across liver tissue shows increasing delocalization with disease progression. The highest intensity is shown in yellow. (E) Lipid zonation becomes less marked in 32‐week WD mice, as determined by principal component scores. Yellow and blue colors represent the most positive and negative scores, respectively. (F) Quantification of the corresponding principal component loadings show that lipid zonation is almost completely lost with NASH.

NASH was induced in WT mice by WD feeding for 32 weeks. The WD contained high levels of cholesterol, sucrose, and saturated fat. A low‐fat diet (LFD) with a similar total fatty acid profile (Supporting Table S4) was used as a control diet. After 12 weeks, the mice being fed a WD developed macro‐ and microvesicular steatosis, in contrast to the mice being fed a LFD (Fig. 4A). After 32 weeks, mice on a WD developed NASH, with characteristic pericentral fibrosis (Fig. 4A), increased serum alanine aminotransferase levels, and transcripts for *Tnf‐α* and *Tgf‐β1* (Fig. 4B).

The hepatic lipid profile was measured by LC‐MS for mice on WD and LFD at 12 and 32 weeks, and the identified lipids were used to construct a PCA model (Fig. 4C). Interestingly, the four groups were clearly distinguished (R^2^ 0.92, Q^2^ 0.88). The most important lipids that distinguished the 32‐week WD mice from the others were TAG(54:3) (18:1/18:1/18:1) and TAG(52:2) (16:0/18:1/18:1), highlighting the importance of increased 18:1 as a marker for both NAFL and NASH (Fig. 4C). We confirmed the increased 18:1 by GC‐MS, measuring total hepatic fatty acid profiles for all samples and their diets (Supporting Fig. S2, Supporting Table S4). The MUFA/SFA ratio and SCD1 activity were substantially increased by WD feeding, with the highest levels determined for the 32‐week WD mice (2‐fold increase).

Next, we examined the zonal distribution of lipids in LFD and WD mice using MALDI‐MSI (Fig. 4D‐F). Consistent with our earlier findings, PC(32:0) and SM(40:1) were associated with the portal vein and central vein, respectively. AA‐containing lipids PC(36:4) and PC(38:4) were located primarily in zone 3 for LFD and 12‐week WD mice, whereas PC(34:1) and PC(32:1) were the major lipids in zone 1 (Fig. 4F). It was extremely difficult to build a statistical model for zonal differences in 32‐week WD mice, however, with zonal distributions for most lipids being relatively weak or absent (Fig. 4E‐4F). This is exemplified by SM(40:1), which is normally associated with central vein (Fig. 4D), becoming increasingly delocalized with disease progression.

### LIPID ZONATION IN HUMAN NAFL AND NASH

**Figure 5:**
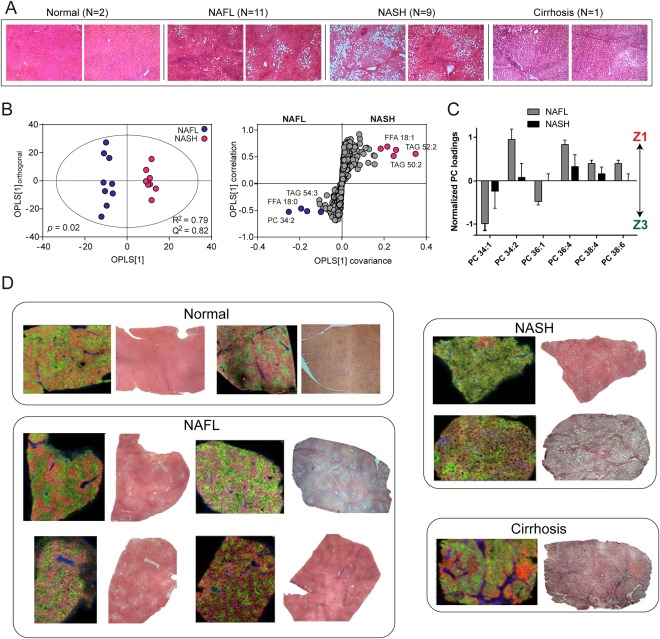
Lipid distributions across the human NAFLD spectrum. (A) H&E‐stained sections of human liver tissue for normal liver, NAFL, NASH, and cirrhosis. Original magnification: × 40. (B) Homogenized liver tissue was analyzed by LC‐MS, and the lipidomic profile was used to compare NAFL and NASH groups, revealing increased shorter chain TAGs and FFA(18:1) in NASH. (C) MALDI‐MSI was performed on frozen sections of tissue and quantified, revealing changes to zonation with NASH. (D) Example MS images of tissue covering the spectrum of NAFLD are shown alongside corresponding adjacent H&E sections. Spatial distributions for PC(32:0) (portal vein), PC(36:4) and PC(34:1) are denoted as blue, red, and green, respectively, for normal liver, NAFL, and NASH. Greater intensity of color reflects relative abundance. Whereas PC(36:4) and PC(34:1) are located in zones 1 and 3, respectively, in both normal and NAFL, there is partial to complete loss of zonation in NASH. Cirrhotic tissue, on the other hand, was marked by lipid changes to fibrotic and nodular regions (Supporting Information). Spatial distributions for lipids denoted in blue, red, and green for cirrhosis are PC(32:0), PC(34:1), and PC(34:2), respectively.

Human liver samples, covering the spectrum of NAFLD from normal to cirrhotic (Fig. 5A), were analyzed using LC‐MS to compare the overall lipid profiles (Supporting Fig. S6). We were specifically interested in the differences between livers presenting NAFL compared to NASH (Fig. 5B). Multivariate analysis of these two groups revealed that samples with NASH had increased FFA(18:1) and short‐chain TAGs, including TAG(52:2) (16:0/18:1/18:1), compared with samples with steatosis only. This was confirmed by GC‐MS (Supporting Table S4) and an increase in SCD1 activity implicated in human NAFL and NASH, which is in excellent agreement with the mouse studies.

We then performed MALDI‐MSI, using adjacent H&E‐stained sections and PCA to identify histological regions and quantify the zonation of lipids (Fig. 5C). Similar to the mouse samples, the portal vein was characterized by PC(32:0), whereas SM(40:1) was found to be associated with both central and portal veins. Interestingly, there was little difference observed between the zonal distributions of lipids in normal and NAFL liver tissue. In both groups, PC(34:2) and PC(36:4) were the most predominant lipids in zone 1, whereas PC(34:1) and PC(36:1) were strongly associated with zone 3 (Fig. 5C,D). With NAFL progression to NASH, however, the differences between pericentral and periportal regions became less pronounced, with partial to complete loss of zonation (Fig. 5C,D). Cirrhotic liver was characterized by nodular and fibrotic regions, rather than the traditional lobular structure (Supporting Fig. S7, Fig. 5D). Areas of fibrosis were characterized by increased PC(32:0) and SM species, whereas nodules had increased PC(34:1) and PC(32:1) (Supporting Fig. S7).

### EICOSANOID METABOLISM WITH NAFLD PROGRESSION

Eicosanoids and lipid mediators are fatty acid metabolites and have been implicated in the inflammation response and in the progression of NAFL to NASH. We therefore extracted these metabolites from homogenized mouse and human liver tissue and analyzed the extracts using LC‐MS/MS (Supporting Fig. S8, Supporting Table S3). Overall, we detected 34 different eicosanoids and lipid mediators, including AA‐derived hydroxyeicosatetranoic acids (HETEs, n = 6), epoxyeicosatrienoic acids (n = 2), dihydroxyeicosatrienoic acids (n = 3), prostaglandins (n = 6); linoleic acid (LA)‐derived hydroxyoctadecadienoic acids (HODEs, n = 2), epoxyoctadecaneoic acids (n = 2), dihydroxyoctadecanoic acids (n = 2); eicosapentaenoic acid (EPA)‐derived hydroxyeicosapentaenoic acids (HEPEs, n = 4) and DHA‐derived hydroxydocosahexaenoic acids (HDoHEs, n = 2).

**Figure 6:**
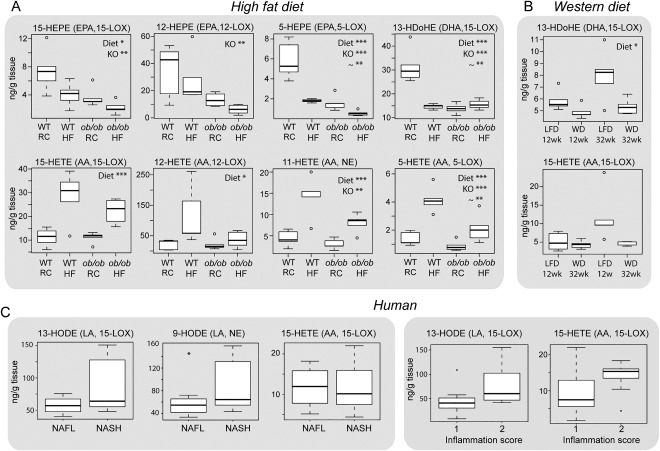
Changes to eicosanoid lipid mediators with NAFLD. Decreased *n‐3* fatty acid (EPA, DHA)‐derived anti‐inflammatory eicosanoids and increased *n‐6* fatty acid (AA)‐derived proinflammatory eicosanoids observed in livers from *ob/ob* mice and mice on an HFD. (A) Two‐way analysis of variance was performed to assess the contribution of the diet, genotype (KO) and their interaction (∼) to the metabolite changes observed. (B) Similarly, a decrease in *n‐3* derived 13‐HDoHE was noted for mice following a WD, whereas no significant difference was determined for the *n‐6*–derived HETEs. Significantly increased *n‐6* LA‐derived proinflammatory HODEs were observed in livers with NASH, compared with livers with NAFL, in human liver tissue. (C) Increased HETEs and HODEs (*P* < 0.1) were observed in human samples with a higher inflammation score. Data are expressed as the median ± upper/lower quartile. *P < 0.05. **P < 0.01. ***P < 0.001.

The resulting hepatic eicosanoid profiles were used to construct OPLS‐DA models to discriminate between WT‐RCD control and NAFL mice (WT‐HFD, *ob/ob*‐RCD, *ob/ob*‐HFD) (Supporting Fig. S9). The most discriminating class of eicosanoids for control and NAFL liver were the HEPEs, HDoHEs, and HETEs (Supporting Fig. S9D). In particular, *n‐3* DHA‐derived 13‐HDoHE and several *n‐3* EPA‐derived HEPEs were significantly decreased in *ob/ob* mice and by HFD feeding (Fig. 6A). In contrast, *n‐6* AA‐derived 12‐ and 15‐HETE were significantly increased by HFD feeding alone, whereas 5‐ and 11‐HETE were significantly increased in *ob/ob* mice and by HFD (Fig. 6A). Next, we assessed the eicosanoid profiles from the WD/LFD mice, finding decreased 13‐HDoHE in WD‐fed mice; however, no significant differences were noted in HETEs (Fig. 6B).

We assessed similar metabolites in human liver and determined that the concentrations of 9‐ and 13‐HODE (which are regulated by LOX and derived from *n‐6* fatty acid LA) were the most discriminating for NAFL and NASH, with both showing increases in NASH (Fig. 6C), whereas there was no difference in 15‐HETE concentration. Given that HETEs and HODEs are proinflammatory metabolites, we further examined how their levels were correlated with the pathologist‐annotated “inflammation score,” finding increased levels with higher scores (*P* < 0.1 for 13‐HODE and 15‐HETE).

### REMODELLING OF PHOSPHOLIPID MEMBRANE FEEDS INTO UP‐REGULATED EICOSANOID SYNTHESIS

**Figure 7:**
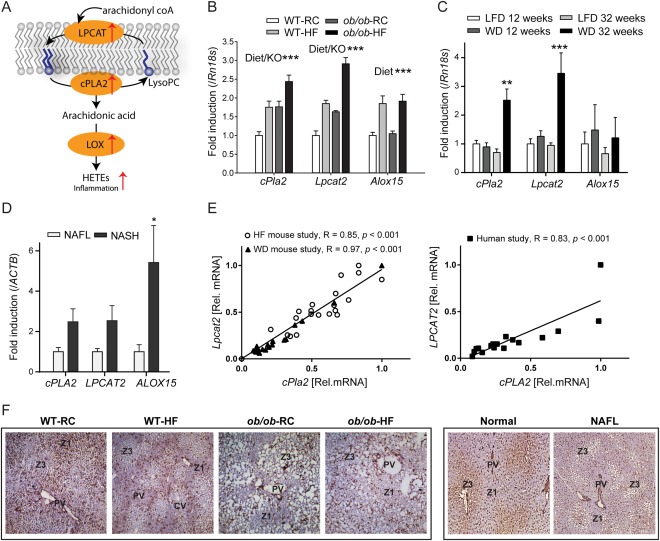
(A) Fatty acid remodeling in NAFLD. Group IVA phospholipase (cPLA2) preferentially cleaves PC to release arachidonic acid (AA). AA can then be metabolized by LOX to form eicosanoids, including proinflammatory HETEs. PCs are regenerated by the action of lysophosphatidycholine acyl transferases (LPCAT) on lysophosphatidylcholines (LysoPC). (B) An increase was observed in hepatic mRNA levels for *cPla2* and *Lpcat2* with HFD and for *ob/ob* mice (two‐way analysis of variance). *Alox15* was significantly increased by HFD‐feeding only. (C) Gene transcripts for *cPla2* and *Lpcat2* were significantly increased in mice fed a Western diet (WD) for 32 weeks. (D) Similarly, hepatic mRNA levels for *cPLA2, LPCAT2*, and *ALOX15* were all increased in human NASH compared with NAFL. *P < 0.05. **P < 0.01. ***P < 0.001. (E) A striking correlation between *Lpcat2* and *cPla2* mRNA levels was noted in both mouse and human studies. (F) Immunostaining showed that LPCAT2 protein has a distinct periportal distribution in WT mice, and a pericentral distribution in *ob/ob* mice and human liver. Original magnification: × 50.

AA is incorporated into the membrane by the action of acyl transferases, notably LPCAT2, whereas cPLA2 preferentially cleaves PC to release free AA, which is then metabolized by LOX to form HETEs and other eicosanoids (Fig. 7A). An increase in AA‐derived HETEs can be therefore be explained by higher amounts of AA‐containing intact lipid in the membrane, increased availability of free AA, increased activity of the LOX pathway, or a combination of these factors. To elucidate which of these pathways are up‐regulated in NAFL, we measured hepatic mRNA levels for *Lpcat2, cPla2, Alox15* (15‐LOX), *Alox5* (5‐LOX), and *Alox12* (12‐LOX) in WT‐RCD control mice and the three corresponding NAFL groups. A significant increase in hepatic mRNA levels for *Lpcat2* and *cPla2* was observed with NAFL in WT‐HFD, *ob/ob*‐RCD, and *ob/ob*‐HFD mice, whereas *Alox15* was significantly increased by high‐fat feeding only (Fig. 7B). No change was noted for *Alox12* or *Alox5* across the groups.

Hepatic transcript levels were measured for the mice fed WD or LFD for 12 and 32 weeks. Interestingly, there was a significant increase in *Lpcat2* (4‐fold) and cPla2 (3‐fold) for the mice that developed NASH (32‐week WD), whereas no change in *Alox15* was observed across groups (Fig. 7C). Similarly, hepatic mRNA levels for *LPCAT2, cPLA2*, and *ALOX15* in human liver samples were measured (Fig. 7D). *LPCAT2* and *cPLA2* were increased by up to 3‐fold in NASH compared with NAFL, whereas *ALOX15* was significantly increased by up to 6‐fold. It is worth noting that human 15‐LOX prefers LA as a substrate producing 13‐HODE, whereas the equivalent murine enzyme acts on AA to generate 15‐HETE. Overall, and despite differences, the agreement between mouse and human data is striking and may suggest a conserved mechanism of NAFLD progression.

## Discussion

We have established the zonal distributions of phospholipids in liver with NAFLD progression, using mouse models of NAFL and NASH, and clinical samples covering a range of NAFLD severity. Overall, both similarities and differences were found between the mouse and human studies. Similar lipids were found associated with portal and central veins, whereas many of the periportal/pericentral zonation patterns were opposite. For instance, PC(34:2) and PC(36:4) are typically zone 3 in mice and zone 1 in humans; PC(34:1) is typically zone 1 in mice and zone 3 in humans. However, in both mouse and human NASH, there was a striking loss in lipid zonation.

Previous studies have shown that knocking out either *Alox15* 37 or *cPla2* 38, 39 in mice resulted in reduced steatosis and inflammation, emphasizing the importance of these pathways in NAFLD. Our data provide evidence at the metabolite and gene expression level for remodeling of the hepatic PC component in NAFLD. Specifically, we show that the activities of LPCAT2, cPLA2, and 15‐LOX are up‐regulated in NAFL and NASH. This results in a cycle of AA enrichment and release from the membrane, generation of proinflammatory eicosanoids, followed by reintroduction of AA into membrane phospholipids (Fig. 7A). Our data further show transcript levels for *LPCAT2* and *cPLA2* to be closely correlated in both the mice studies and the human study, suggesting that these two genes may be coregulated (Fig. 7E). Phospholipid remodeling in obesity has been linked to ER stress and disrupted calcium signalling.^40^ Given that the activities of both LPCAT2 and cPLA2 are calcium‐dependent, further elucidation of the relationships between ER stress, calcium signaling, and lipid remodeling in NAFLD may be warranted.

Of particular interest in this study is LPCAT2, which reintroduces PUFAs—notably AA—into the membrane. Using immunohistochemistry, we found higher staining for LPCAT2 protein in zone 1 for WT mice and in zone 3, particularly around regions of steatosis, for *ob/ob* mice (Fig. 7F). The specific zonation of LPCAT2 protein, in addition to its increased gene expression, may account for the enrichment of AA‐containing PCs in zone 3, which was noted in the MSI experiments for murine NAFL. In contrast, LPCAT2 protein is pericentrally located in normal and NAFL human liver (Fig. 7F). Because AA‐containing PCs are typically periportal in human liver, an enrichment of LPCAT2 and AA in zone 3 would contribute to a loss of zonation for this lipid, which we observed in NASH. Although it is unclear whether the change in lipid zonation is a cause or effect of NAFLD, there does appear to be a link.

As a result of enrichment of AA in zone 3, generated eicosanoids will provide an inflammatory insult to hepatocytes in the pericentral region, in particular. This may account for the increased oxidative damage observed in this region during NASH. Furthermore, because LPCAT2 is induced by proinflammatory cytokines^41^, ^42^ and is highly expressed in inflammatory cells such as macrophages and neutrophils,^41^ this could further reinforce the inflammation cascade. We stained for CD68‐positive cells (Kupffer cells, macrophages, monocytes; Supporting Fig. S10) and found that these cells were distributed azonally in normal and NAFL human liver but in NASH were pericentrally located (also discussed in Lefkowitch et al.^43^). Similarly, in human and murine NASH, LPCAT2 staining was highest surrounding pericentrally located lipid droplets (Supporting Figs. S11, S12) and inflammatory infiltrates. Taken together, this leads us to speculate that lipid droplet biology and macrophage infiltration may be important factors in the lipid remodeling observed during NAFLD.

Overall, this study describes the location of lipids and their associated metabolic pathways in NAFL and NASH. We provide a link between the up‐regulation of enzymes involved in membrane remodeling, fatty acid release, eicosanoid formation, and lipid composition while suggesting areas of interest for future study. The integration of mass spectrometry imaging technology with computational tools and traditional biochemical techniques has been shown here as a powerful approach. Importantly, this has enabled the fatty acid content of intact phospholipids to be mapped to particular locations in tissue and presents the opportunity to follow changes to lipid composition and distribution with disease state.

## Supporting information

Additional Supporting Information may be found at onlinelibrary.wiley.com/doi/10.1002/hep.28953/suppinfo.

Supporting Information
