# Supplementary material for: Lipid zonation and phospholipid remodeling in nonalcoholic fatty liver disease
Source: Hepatology. 2017 Feb 6;65(4):1165–80. doi: 10.1002/hep.28953 (PMC5396354; doi:10.1002/hep.28953)
Supplement: Supplementary file 1 — Supporting Information [file HEP-65-1165-s001.pdf]

Supporting Information - HEP-16-0510

Lipid zonation and phospholipid remodeling in non-alcoholic fatty liver disease

Zoe Hall[1,2], Nicholas J. Bond[2], Tom Ashmore[1], Francis Sanders[2], Zsuzsanna Ament[2], Xinzhu Wang[1], Andrew J. Murray[3], Elena Bellafante[4], Sam Virtue[5], Antonio Vidal-Puig[5], Michael Allison[6], Susan E. Davies[7], Albert Koulman[2], Michele Vacca[1, 2, 5], Julian L. Griffin[1,2,*]

[1] Department of Biochemistry and Cambridge Systems Biology Centre, University of Cambridge, 80 Tennis Court Road, Cambridge, UK

[2] MRC Human Nutrition Research, 120 Fulbourn Road, Cambridge, UK

[3] Department of Physiology, Development and Neuroscience, University of Cambridge, Downing Street, Cambridge, UK

[4] Mario Negri Sud, Santa Maria Imbaro, Chieti, Italy

[5] Metabolic Research Laboratories, Wellcome Trust-MRC Institute of Metabolic Science, Addenbrooke's Hospital, University of Cambridge, UK

[6] Liver Unit, Department of Medicine, Cambridge University Hospitals NHS Foundation Trust, Cambridge, UK

[7] Department of Histopathology, Cambridge University Hospitals NHS Foundation Trust, Cambridge, UK

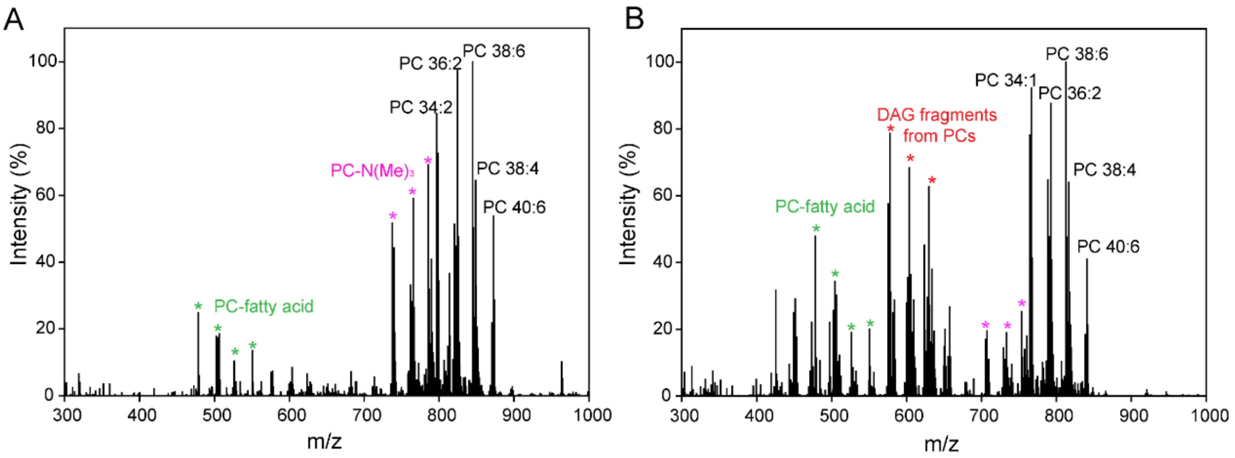

Figure S1. Representative average MALDI-MSI spectra. A typical mass spectrum for mouse liver tissue with potassium nitrate (**A**) or lithium nitrate wash (**B**). Spectra are predominantly characterised by the corresponding K^+ or Li^+ phosphatidylcholine adducts, and their in-source fragment ions (green, pink and red asterisks). Li^+ adducts are more prone to fragmentation, and hence useful for identification of lipids by tandem MS.

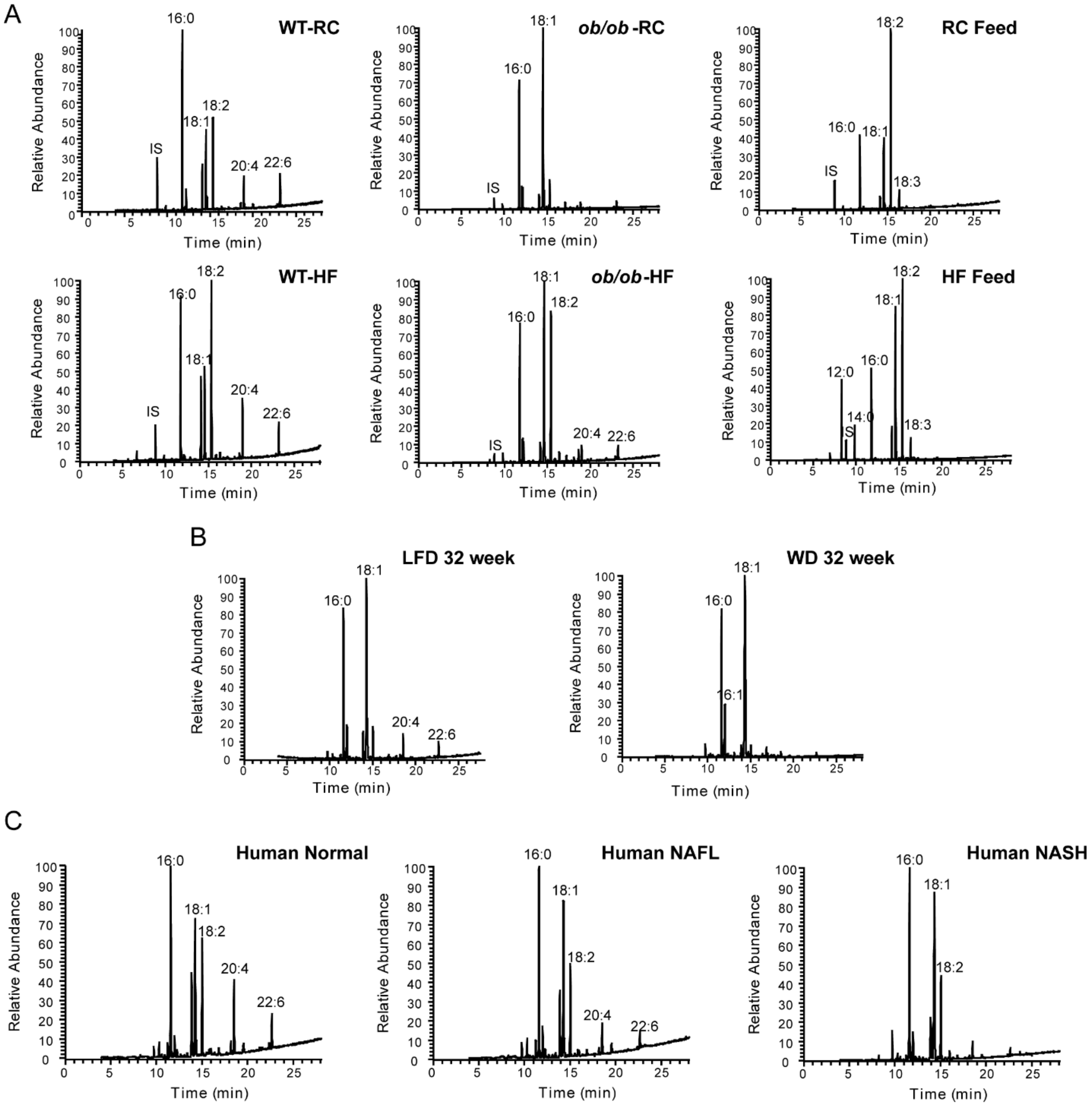

Figure S2. Representative GC-MS chromatograms. Total fatty acids (FAMEs) were compared for wild type (WT) and *ob/ob* mice fed a high fat (HF) or regular chow (RC) diet, and compared to their respective feeds (**A**). Total fatty acid profiles for WT mice on a Western diet (WD) or low fat diet (LFD) after 32 weeks feeding (**B**). Example GC-MS fatty acid profiles for human samples (**C**). Full results are in Table S4.

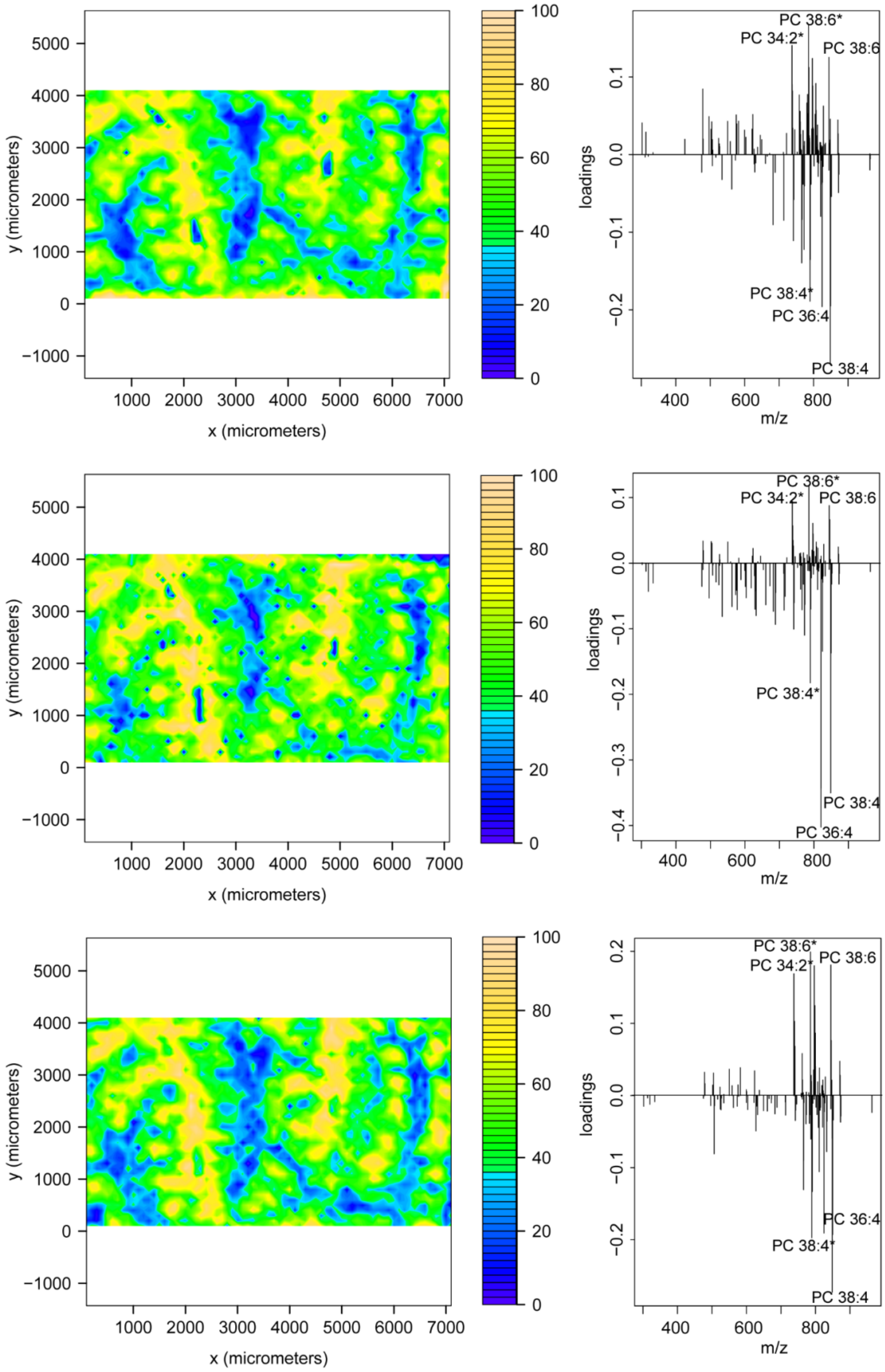

Figure S3. Assessing method reproducibility. We measured the zonal distribution of lipids using MALDI-MSI across serial sections of *ob/ob*-RC liver, finding excellent agreement in zonation distributions of lipids and their relative loadings scores. Species annotated with asterisk have been identified as fragments of intact lipids.

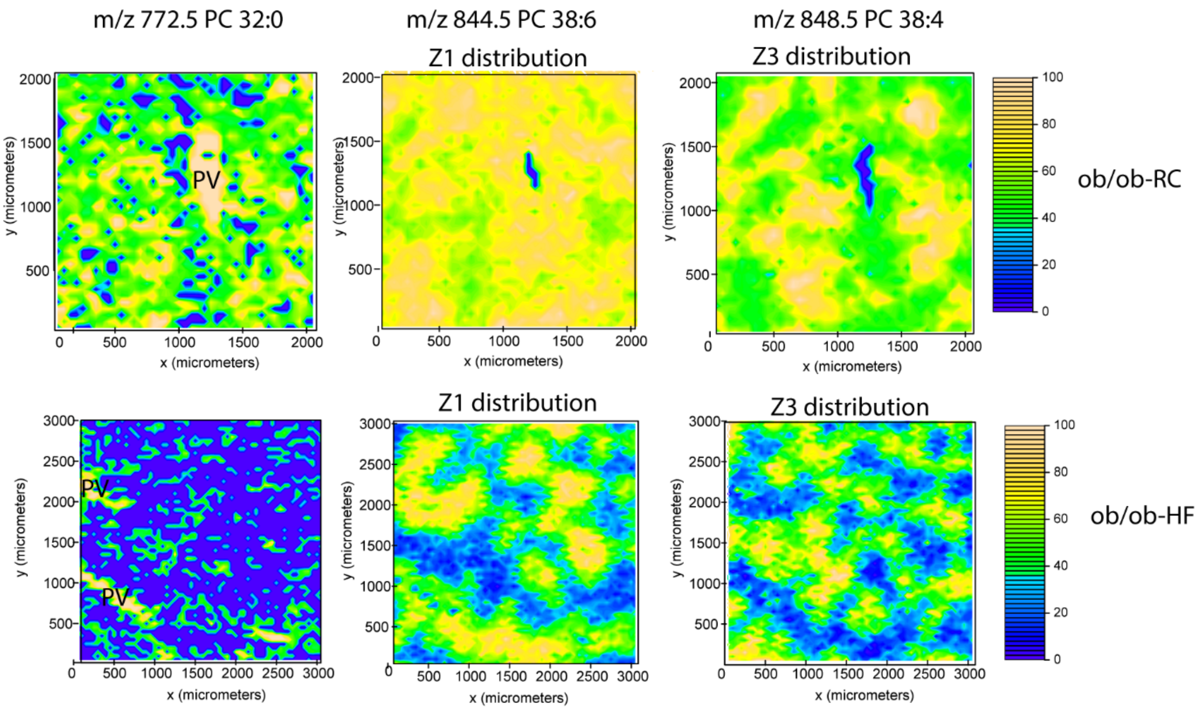

Figure S4. Zonal distributions of key lipid across liver tissue measured by MALDI-MSI for *ob/ob*-RC and *ob/ob*-HF mice. PC(32:0) is associated with the portal vein, whereas PC(38:6) and PC(38:4) have predominantly zone 1 and zone 3 distributions respectively. High similarity between the two groups was observed in terms of lipid zonation.

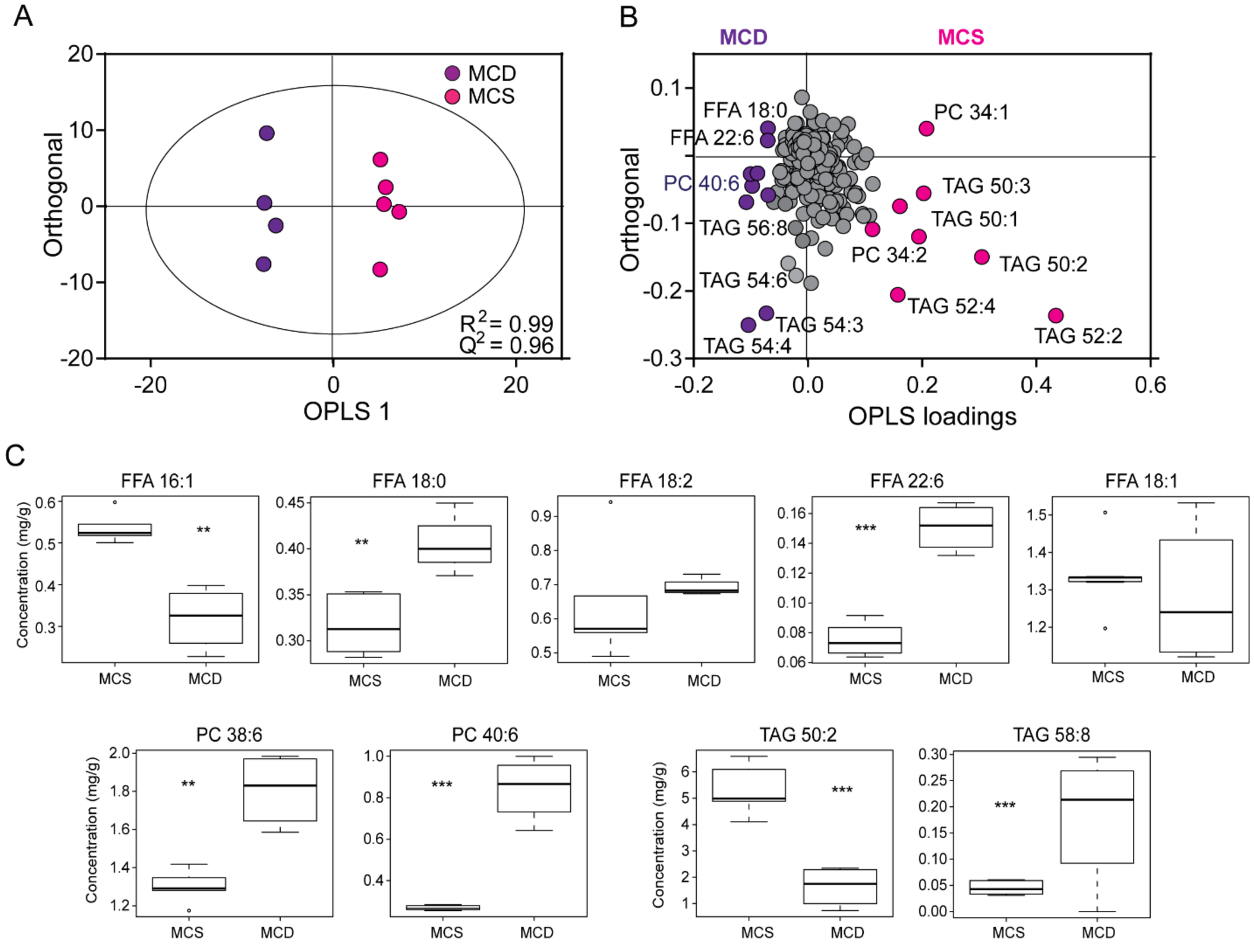

Figure S5. Hepatic lipid signatures for MCD diet mice. We analysed liver tissue by LC-MS from mice on methionine choline deficient (MCD) and methionine choline sufficient (MCS) control diets. We determined that the main differences were arising from increased PUFA content in MCD mice – specifically lipids containing 22:6 (PC(38:6); PC(40:6); TAG(58:8)) and free fatty acid 22:6. There was no difference found for free 18:1 – proposed as a marker for NAFL and NASH. The lipid profile for the MCD mice was highly dissimilar to that observed for the other two mouse studies (high fat diet; Western diet), and from the clinical samples with either NAFL or NASH diagnosis. We therefore concluded that the MCD diet was not suitable for studying lipid profile changes representative of human NASH. This may be a result of lipolysis of adipose tissue, which is a potential effect of the MCD diet resulting in loss of body weight. This could consequently alter the hepatic lipid profile through the increased circulation of PUFA from adipose tissue (1). Additionally, the MCD diet by its nature directly affects the metabolism of phosphatidylcholine lipids (since there is a deficiency of choline) and this again may render it unsuitable for a lipidomic study. In contrast, feeding mice with a Western diet for 32 weeks resulted in mice with NASH and metabolic features matching human NASH (increased body weight, insulin resistance, increased 18:1 and shorter chain TAGs).

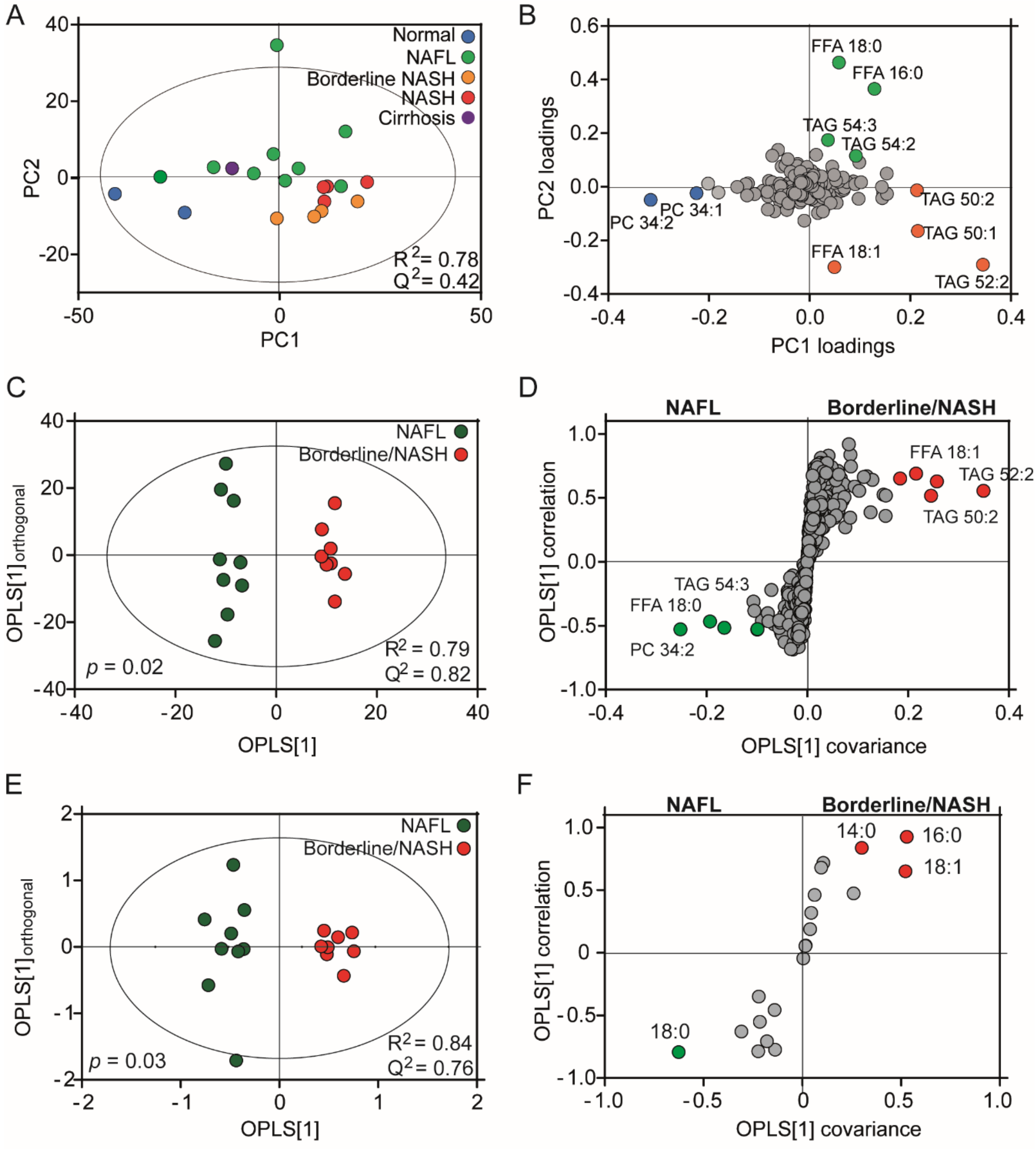

Figure S6. Lipid signatures in liver tissue change with human NAFLD progression. LC-MS was used to compare the lipid profiles across samples, and a PCA model constructed (**A**), which clearly distinguishes liver tissue with borderline NASH/NASH from the other samples principally based on increased FFA(18:1) and TAGs with shorter fatty acid chains, e.g. TAG(52:2) (16:0/18:1/18:1) (**B**). An OPLS-DA model (**C**) was built to compare NAFL with NASH liver. From the OPLS-DA model, an S-plot (**D**) was constructed (2), which combines the modelled covariance and modelled correlation from the OPLS-DA model in a scatter plot. This enabled the determination of the most important and reliable lipid species for distinguishing the two groups (right upper and left lower quadrants for NASH and NAFL tissue respectively). Similarly GC-MS analysis on total fatty acid profiles revealed an increase in *de novo* lipogenesis products 16:0 and 14:0 and MUFA 18:1 in NASH compared to NAFL (**E-F**).

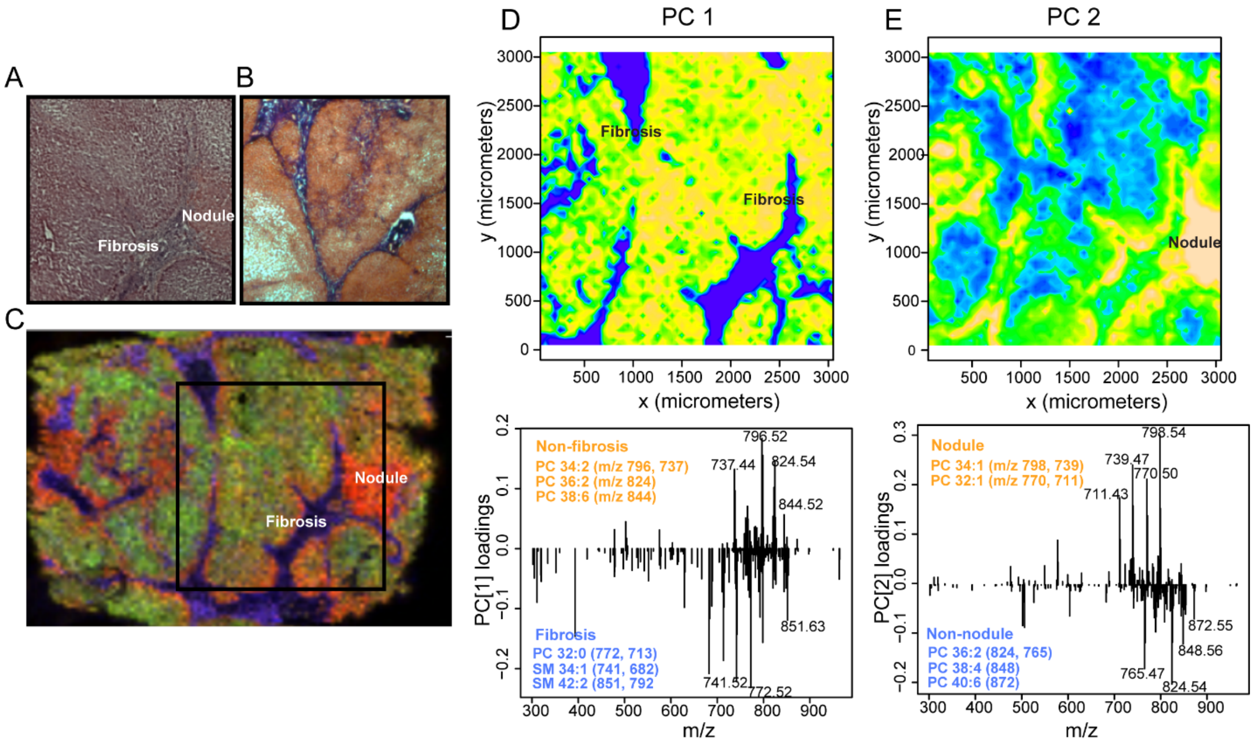

Figure S7. Principal components analysis (PCA) of cirrhotic human liver tissue. Sections of liver tissue with cirrhosis are shown stained with H&E (**A**) or Masson's trichrome stain (**B**), in which fibrosis is stained blue. Two-dimensional ion distributions obtained by MALDI-MSI are shown (**C**) for PC(32:0) K^+ (*m/z* 772.52, blue), PC(34:1) K^+ (*m/z* 798.54, red) and PC(34:2) K^+ (*m/z* 796.52, green). PCA scores and loadings plots are given for the first two principal components (**D**, **E**). Yellow and blue in contour plots represent most positive and negative scores, respectively, corresponding to non-fibrotic and fibrotic regions (**D**) and nodule and non-nodule regions (**E**). Fibrotic regions are characterised by increased SM lipids and PC 32:0, whereas the nodular area has increased PC 34:1 and PC 32:1.

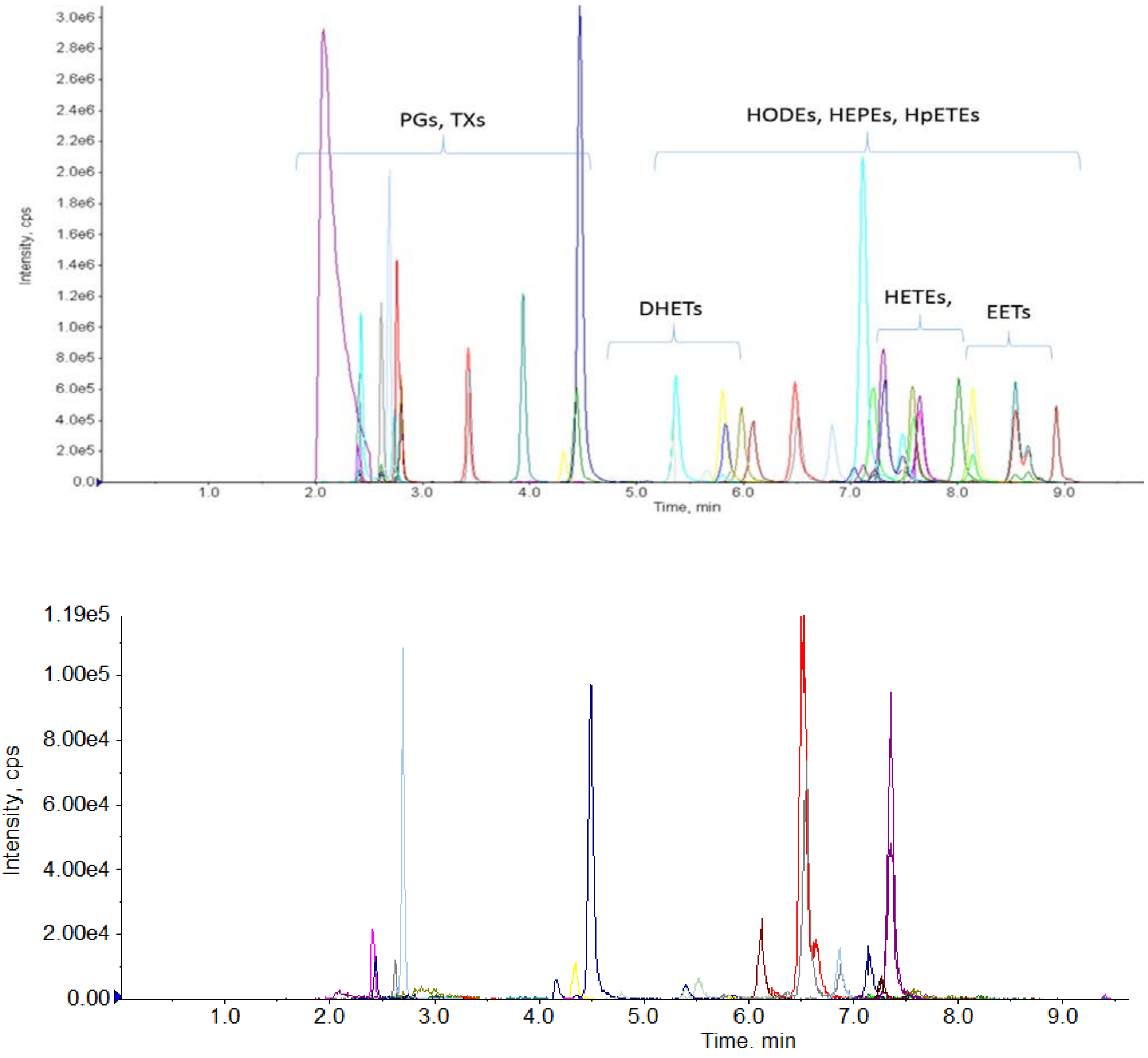

Figure S8. Extracted multiple reaction monitoring (MRM) chromatograms for standard mix (100 nM) of eicosanoids (top) and typical mouse liver sample with NAFL (bottom). Region of chromatographic elution for major classes of eicosanoids are highlighted for prostaglandins (PGs), thromboxanes (TXs), dihydroxyeicosatrienoic acids (DHETs), hydroxyeicosatetraenoic acids (HETEs), epoxyeicosatrienoic acids (EETs), hydroxyoctadienoic acids (HODEs), hydroxyeicosapentaenoic acids (HEPEs), hydroperoxy-eicosatetraenoic acids (HpETEs).

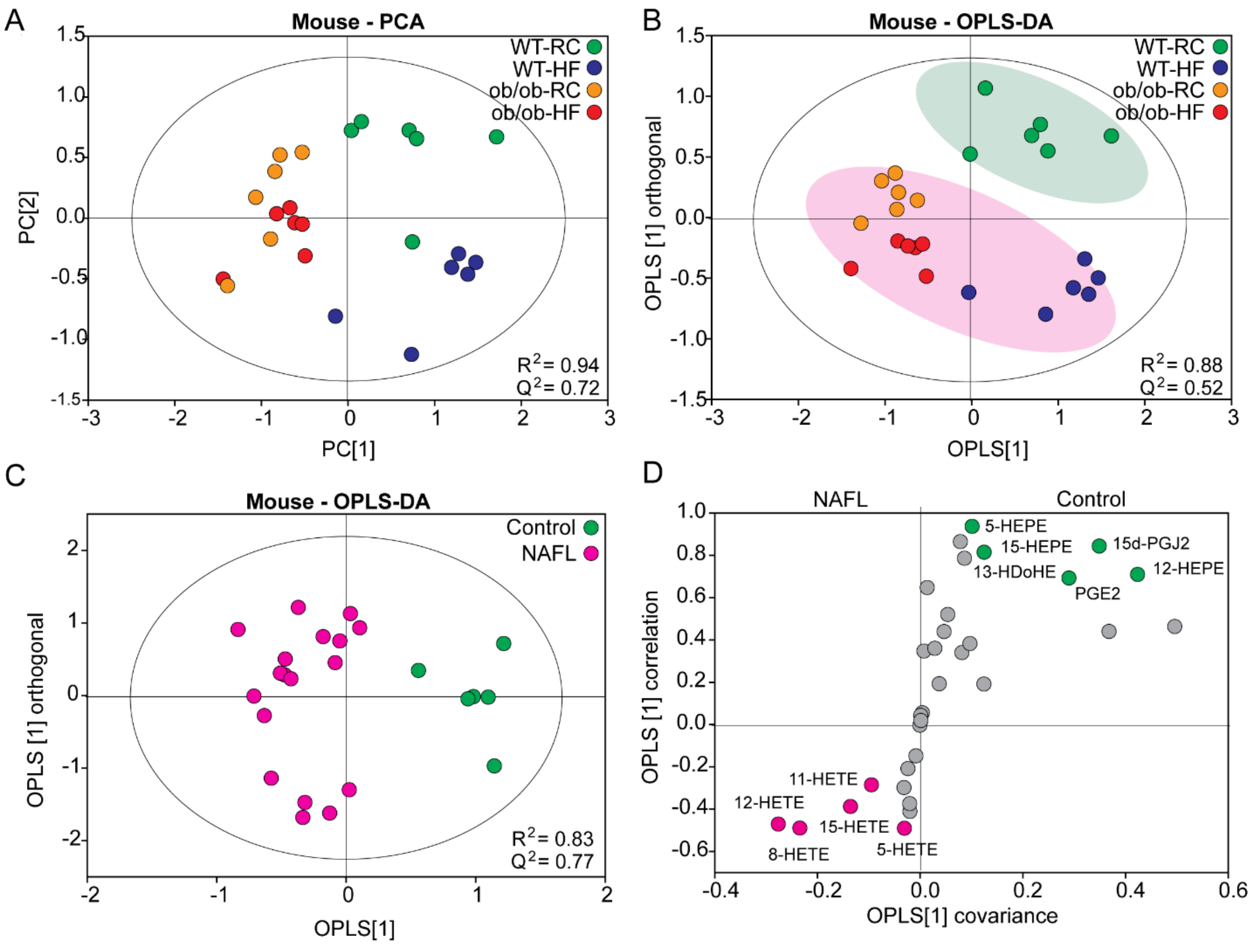

Figure S9. Eicosanoid profiles in murine NAFL. Eicosanoids were measured in homogenised mouse liver and eicosanoids profiles used to construct PCA (**A**) and OPLS-DA models (**B**). Of particular interest was the major differences in eicosanoids between control (n=5) and NAFL (n=15) liver tissue (**C**). Using this model, we constructed an S-plot, which combines the modelled covariance and modelled correlation from the OPLS-DA model in a scatter plot (**D**). This enabled the determination of the most important and reliable eicosanoid species for distinguishing the two groups (right upper and left lower quadrants for control and NAFL tissue respectively).

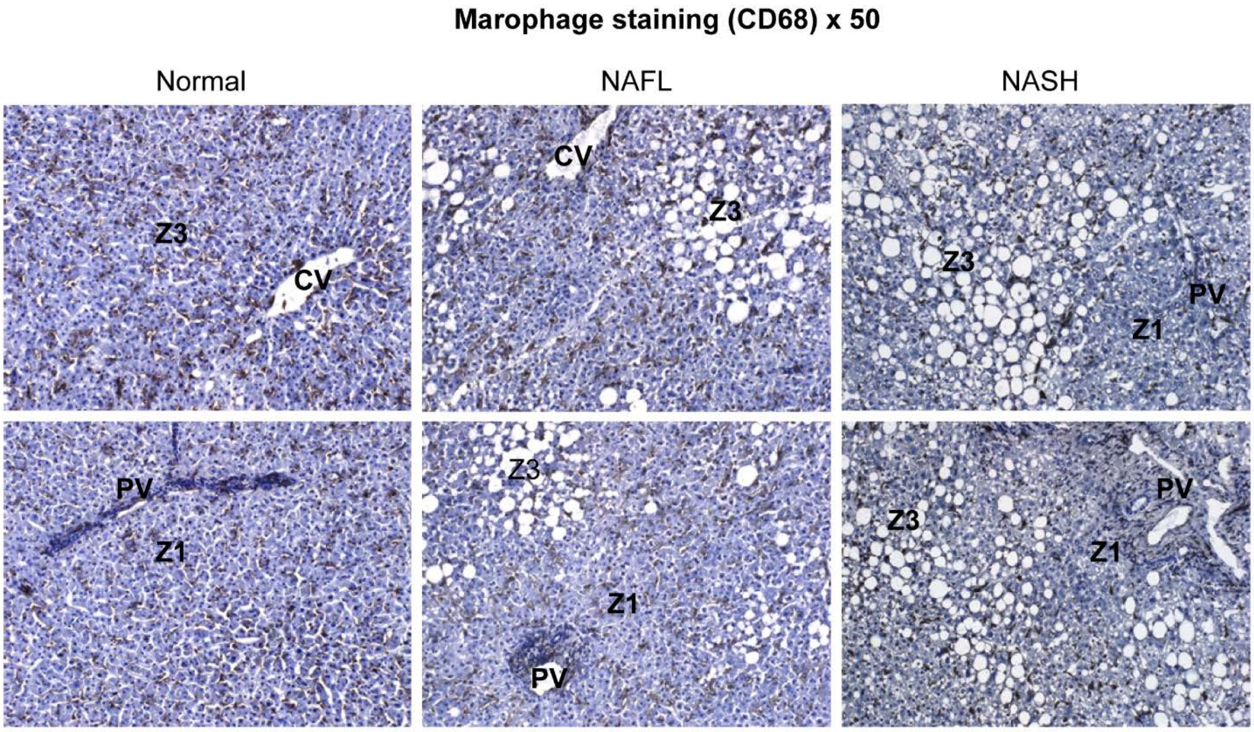

Figure S10. Immunostaining for CD68 positive cells in human liver. CD68 positive cells (marker for macrophages including Kupffer cells) appear to be distributed azonally in human normal and NAFL liver. In human NASH, there appears to be increased staining (brown) around lipid droplets in the steatotic regions (zone 3).

LPCAT2 staining in human NASH

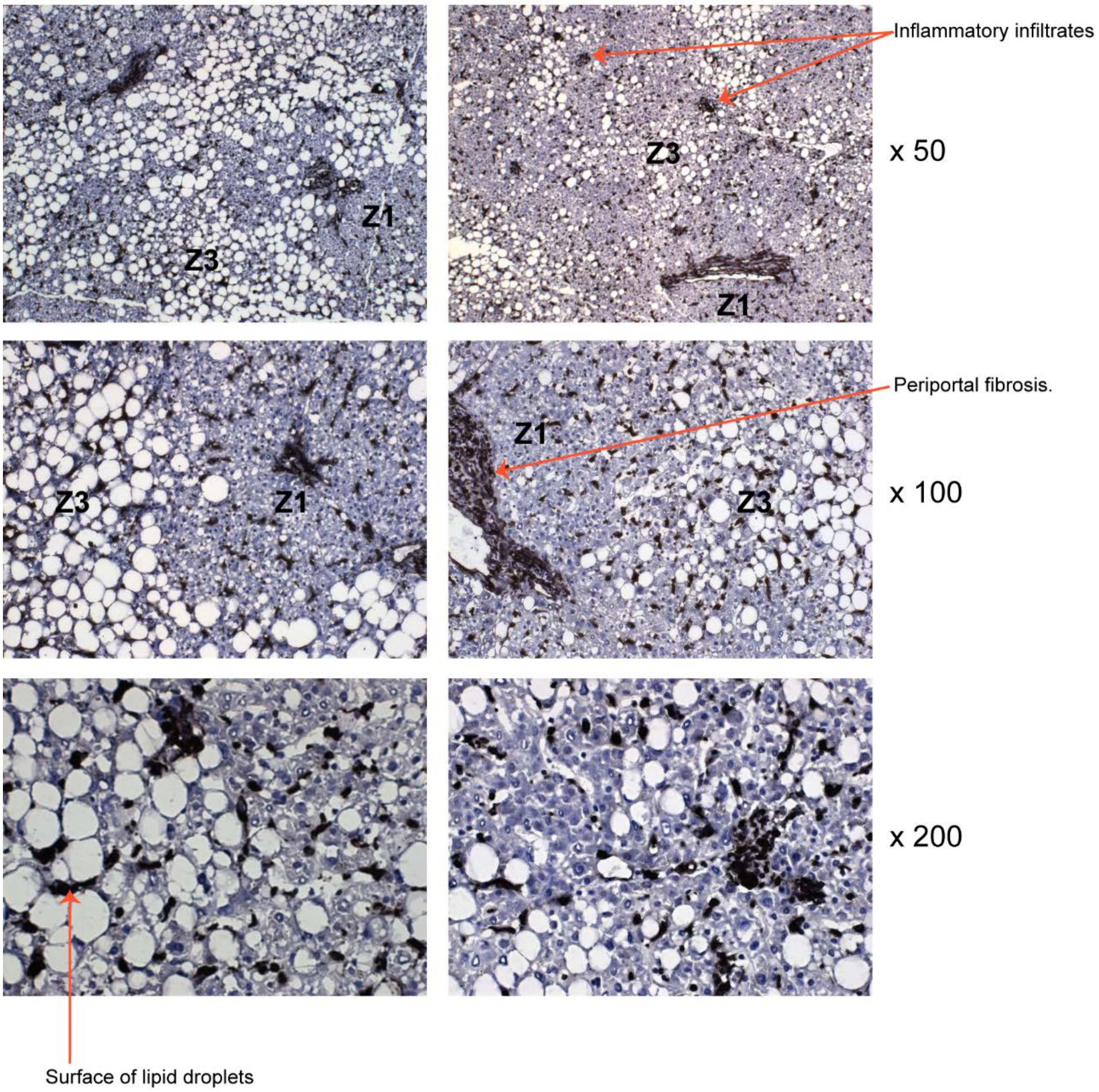

Figure S11. LPCAT2 staining in human NASH. LPCAT2 staining in human NASH appears to be associated with the surface of lipid droplets (mainly Z3), as well as inflammatory infiltrates, and areas of periportal fibrosis.

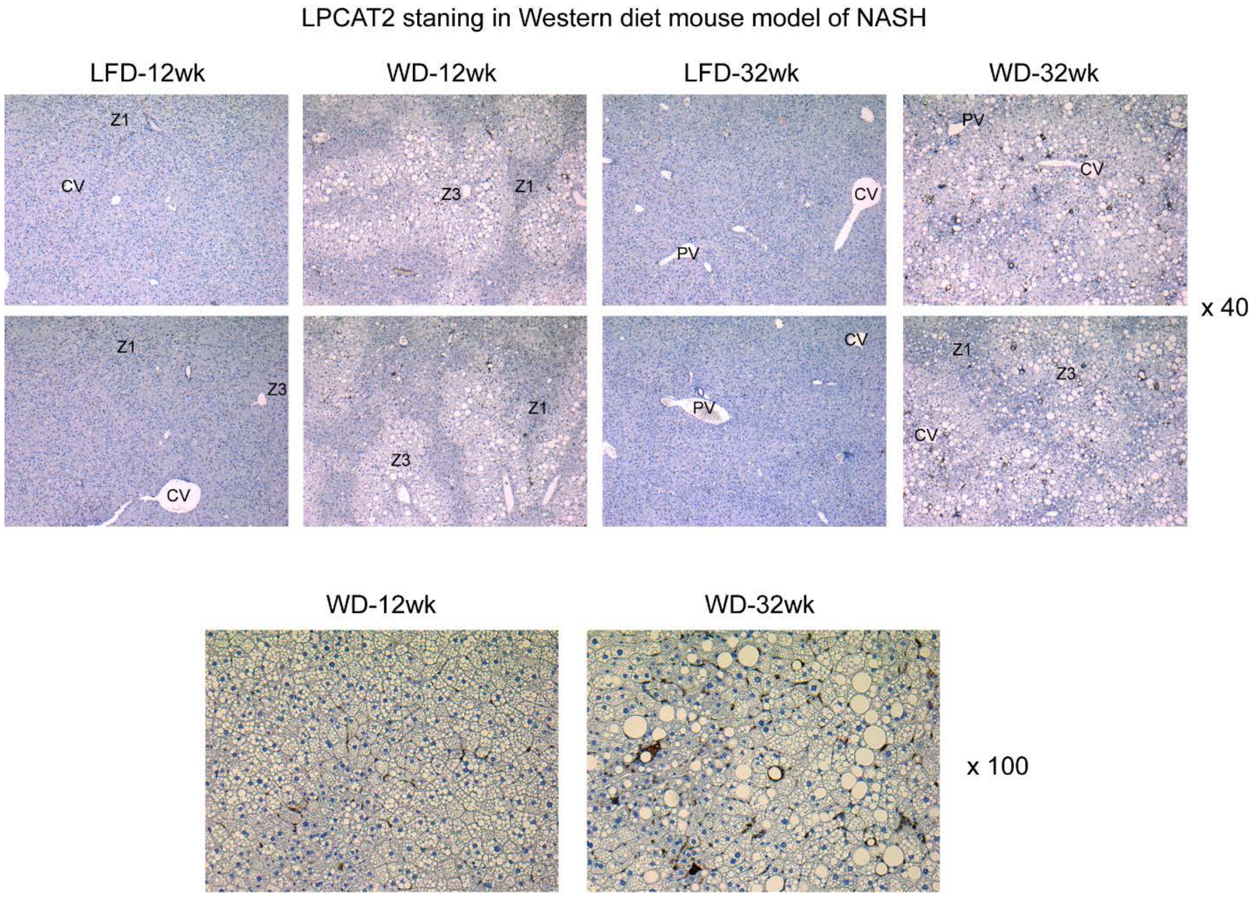

Figure S12. LPCAT2 staining in a mouse model of NASH. LPCAT2 staining in low fat diet (LFD) control mice (12 and 32 weeks) and 12 week Western diet (WD) mice was weak, mainly associated with Kupffer cells, showing a periportal pattern of zonation (similar to that noted for WT mice on regular chow or high fat diets). However, mice on WD for 32 weeks with NASH had dramatically increased staining for LPCAT2, which was often associated with the surface of lipid droplets (as noted in human NASH).

Table S1. Chromatography gradient composition for lipidomics open profiling

Time (min)	Mobile Phase A (%)	Mobile Phase B (%)
0	60	40
0.8	57	43
0.9	50	50
4.8	46	54
4.9	30	70
5.8	19	81
8	1	99
8.5	1	99
8.6	60	40
10	60	40

Table S2. Chromatography gradient composition for targeted eicosanoid analysis

Time (min)	B (%)	Time (min)	B (%)
0.0	15	5.6	60
0.4	15	8	70
0.8	30	8.5	80
1.9	47	10.1	100
2.7	54	10.3	15
3.2	55	12	15

Table S3. MRM transitions and retention times for eicosanoids. Mass transitions (Q1/Q3), collision energies (CE) in volts and retention times (RTs) in minutes of eicosanoid analytes measured.

Eicosanoid	Q1	Q3	RT	CE	Eicosanoid	Q1	Q3	RT	CE
13-HODE	295.0	195.0	6.72	-24	PGJ2	333.0	233.0	3.52	-16
12(13)-EpOME	295.1	195.1	8.22	-22	12-oxoLTB4	333.0	179.0	4.59	-18
9-HODE	295.1	171.1	6.75	-26	PGB2	333.0	235.0	3.53	-26
9(10)-EpOME	295.1	171.1	8.34	-24	12-HpETE	335.0	273.0	7.46	-12
9,10-DHOME	313.1	201.0	4.62	-28	LTB4	335.1	195.0	4.04	-22
12,13-DiHoME	314.1	129.0	4.47	-30	15-HpETE	335.1	113.0	7.47	-14
15d-PGJ2	315.0	271.0	5.88	-20	11,12-DHET	337.0	145.0	5.19	-26
15-oxo-ETE	317.0	273.0	7.43	-20	8,9-DHET	337.1	127.1	5.47	-28
12-oxo-ETE	317.0	153.0	8.1	-22	5,6-DHET	337.1	145.0	5.91	-24
12-HEPE	317.1	179.1	6.32	-20	14,15-DHET	337.1	126.9	4.85	-28
8-HEPE	317.1	155.1	6.22	-18	13-HDoHE	343.0	193.0	7.37	-16
5-HEPE	317.1	115.1	6.57	-18	17-HDoHE	343.1	201.2	7.28	-20
15-HEPE	317.1	219.1	6.06	-18	7-HDoHE	343.1	281.4	7.69	-18
8,9-EET	319.0	167.0	8.72	-18	PGE2	351.1	271.1	2.93	-24
5,6-EET	319.0	163.0	8.83	-20	PGD2	351.1	189.0	2.87	-28
20-HETE	319.0	275.0	6.14	-22	PGF2a	353.0	113.0	2.72	-34
15-HETE	319.0	219.0	7.07	-18	PGD1	353.1	317.0	2.9	-18
5-HETE	319.0	115.0	7.89	-18	PGE1	353.1	112.9	2.71	-32
11,12-EET	319.1	167.2	8.61	-20	8-iso-PGF2a	353.1	193.2	2.45	-36
8-HETE	319.1	301.2	7.54	-18	10,17 DiHDoHE	359.1	153.0	4.08	-22
11-HETE	319.1	167.1	7.35	-22	6-keto-PGF1a	369.1	163.1	2	-38
14,15-EET	319.1	219.2	8.23	-18	TxB2	369.1	169.0	2.48	-24
9-HETE	319.1	151.0	7.71	-20	PGD2-EA	394.5	276.0	2.3	-14
14(15)EpETE	319.1	113.0	8.2	-22	LTD4	494.8	177.0	3.08	-28
12-HETE	319.2	179.2	7.54	-20	12-HETE-d8	327.0	184.0	7.47	-22
15-oxoEDE	321.1	113.1	8.64	-30	PGE2-d4	355.3	275.3	2.76	-28
15-HETrE	321.1	303.1	7.97	-20	8-iso-PGF2a-d4	357.2	197.4	2.45	-34

Abbreviations: dihydroxyeicosatrienoic acid (DHET), dihydroxyoctadecaenoic acid (DiHOME), epoxyeicosatrienoic acid (EET), epoxyeicosatetraenoic acid (EpETE), epoxyoctadecaenoic acid (EpOME), hydroxydocosahexaenoic acid (HDoHE), hydroxyeicosatrienoic acid (HETrE), hydroxyeicosapentaenoic acid (HEPE), hydroxyeicosatetraenoic acid (HETE), hydroxyoctadecadienoic acid (HODE), hydroperoxyeicosatetraenoic acid (HpETE), leukotriene (LT), oxoeicosatetraenoic acid (oxo-ETE), oxoeicosadienoic acid (oxoEDE), prostaglandin (PG), thromboxane (TX)**.**

Table S4. Total fatty acid content (relative %) measured by GC-MS for the two mouse studies and their feeds, and the clinical samples. Total saturated fatty acids (SFA), monounsaturated fatty acids (MUFA) and polyunsaturated fatty acids (PUFA) are given for each group along with the SCD1 ratio (3): (18:0+16:0/18:1+16:1).

Lipid	*High fat diet mouse study*						*Western diet mouse study*					*Human study*		
species	WT-RC	ob/ob-RC	WT-HF	ob/ob-HF	RCD feed	HFD feed	LFD-12wk	LFD-32wk	WD-12wk	WD-32wk	WD/LFD feed	NAFL	Borderline	NASH
**12:0**	0.0	0.0	0.2	0.1	0.0	7.7	0.2	0.1	0.1	0.1	3.3	0.4	0.4	0.4
**13:0**	0.0	0.0	0.0	0.0	0.0	0.0	0.1	0.0	0.0	0.0	0.0	0.5	0.2	0.2
**14:0**	0.4	0.7	0.6	0.9	0.6	4.0	0.8	1.1	1.6	1.7	10.6	1.9	2.6	3.1
**15:0**	0.2	0.1	0.2	0.1	0.1	0.1	0.2	0.2	0.4	0.3	0.0	0.2	0.3	0.4
**16:0**	30.8	29.1	20.3	22.9	16.6	13.4	29.0	27.9	28.3	26.4	28.9	27.1	30.2	29.6
**16:1**	2.8	4.5	0.7	3.1	0.6	0.2	6.8	6.5	11.6	10.0	1.5	3.3	5.2	3.9
**17:0**	0.3	0.1	0.4	0.2	0.1	0.1	0.2	0.2	0.2	0.2	0.0	0.3	0.3	0.4
**17:1**	0.2	0.2	0.2	0.1	0.1	0.1	0.4	0.4	1.0	0.9	0.0	0.4	0.4	0.4
**18:0**	11.3	3.5	11.9	3.1	3.2	5.9	8.5	6.0	2.8	2.2	12.5	12.5	8.7	7.9
**18:1**	14.0	49.3	16.2	35.7	19.3	29.4	28.9	41.8	45.1	50.9	24.9	29.1	31.9	34.9
**18:2 trans**	0.1	0.3	0.1	0.1	0.1	0.0	0.3	0.3	0.4	0.1	1.3	0.3	0.3	0.3
**18:2 cis**	20.2	6.2	28.6	23.6	51.4	34.9	9.7	6.2	3.6	2.5	2.3	11.9	10.8	11.7
**18:3 n6**	0.3	0.1	0.8	0.5	0.3	0.2	0.3	0.2	0.2	0.2	0.7	0.4	0.4	0.5
**18:3 n3**	0.5	0.3	1.3	1.1	4.9	3.5	0.2	0.2	0.1	0.1	0.0	0.7	0.8	0.9
**20:1**	0.4	1.4	0.6	0.9	0.7	0.2	0.7	1.1	1.4	2.0	0.0	1.8	1.0	0.8
**20:2**	0.3	0.1	0.4	0.5	0.1	0.0	0.2	0.3	0.1	0.2	0.0	0.2	0.2	0.1
**20:3 n6**	1.2	0.6	1.3	1.7	0.1	0.0	1.3	0.7	0.5	0.3	0.0	1.0	0.6	0.4
**20:4 n6**	7.7	1.4	9.8	2.9	0.1	0.0	7.4	4.1	1.4	0.9	0.0	3.7	2.9	1.9
**20:5 n3**	0.8	0.2	0.4	0.3	0.5	0.0	0.6	0.3	0.4	0.4	0.0	2.8	1.4	1.1
**22:6 3**	8.4	1.7	6.0	2.2	1.0	0.0	4.2	2.5	0.8	0.6	0.0	1.5	1.4	1.0
**SFA**	42.75	33.45	33.18	27.13	20.65	31.16	38.76	35.35	33.19	30.77	61.80	42.66	42.31	41.55
**MUFA**	17.68	55.61	18.12	39.95	20.75	29.99	37.03	49.95	59.31	64.08	27.30	34.95	38.84	40.59
**PUFA**	39.11	10.46	48.17	32.26	58.31	38.76	23.71	14.19	7.00	4.82	4.70	21.96	18.36	17.46
SCD1 ratio	*0.40*	*1.65*	*0.53*	*1.49*			*0.95*	*1.42*	*1.83*	*2.13*		*0.82*	*0.95*	*1.04*

Table S5. Identification of lipid species detected in mouse and human liver tissue by MALDI-MSI.

Lipid species	*m/z* (K+)	*m/z* (Li+)	Exact mass (K+)	Exact mass (Li+)	ppm (K+)	ppm (Li+)	Fatty acid composition	Confirmed by MS/MS	Diagnostic fragment ions (Li+ precursor)			
									Loss of FA (H+)	Loss of FA (Li+)	Loss of $N(Me)_3$	DAG (H+)
PC 32:0	772.523	740.574	772.5253	740.5776	3.2	4.7	16:0/16:0	Yes	478.3	484.3	681.5	557.5
PC 32:1	770.507	738.562	770.5097	738.562	3.1	0.1	16:0/16:1	Yes	478.3	484.3	679.5	555.5
PC 34:1	798.538	766.590	798.541	766.5933	3.9	4.8	16:0/18:1	Yes	478.3, 504.3	484.3, 510.3	707.5	577.5
PC 34:2	796.522	764.574	796.5253	764.5776	4.0	5.0	16:0/18:2	Yes	478.3, 502.3	484.3, 508.3	705.5	575.5
PC 34:3	794.508	762.562	794.5097	762.562	2.3	0.1	16:1/18:2					
PC 36:1	826.574	794.628	826.5723	794.6246	-1.5	-4.2	18:0/18:1	Yes	506.3	512.3, 510.3	735.5	611.5
PC 36:2	824.553	792.605	824.5566	792.6089	4.1	4.8	18:1/18:1 and 18:0/18:2	Yes	502.3, 504.3, 506.3	508.3, 510.3, 512.3	733.5	609.5
PC 36:3	822.538	790.590	822.541	790.5933	3.6	4.4	16:0/20:3 and 18:2/18:1	Yes	478.3, 502.3, 504.3, 528.3	484.3, 508.3, 510.3, 534.3	731.5	607.5
PC 36:4	820.522	788.574	820.5253	788.5776	4.0	5.1	16:0/20:4	Yes	478.3, 526.3	484.3, 532.3	729.5	605.5
PC 38:3	850.571	818.627	850.5723	818.6246	1.4	-3.1	18:0/20:3					
PC 38:4	848.553	816.605	848.5566	816.6089	4.1	4.8	18:0/20:4	Yes	506.4, 526.3	512.4, 532.3	757.5	633.5
PC 38:6	844.522	812.574	844.5253	812.5776	4.3	5.0	16:0/22:6	Yes	478.3, 550.3	484.3, 556.3	753.3	629.5
PC 40:6	872.553	840.605	872.5566	840.6089	4.1	4.8	18:0/22:6	Yes	506.3, 550.3	512.4, 556.3	781.5	657.5
PC 40:7	870.537	838.589	870.5410	838.5933	4.3	5.1	18:1/22:6	Yes	504.3, 550.3	510.4, 556.3	779.5	655.5
LysoPC16	534.294	502.346	534.2956	502.3479	3.4	4.0	16:0				443.3	
SM 34:1	741.529	709.583	741.5307	709.583	2.0	-0.3		Yes			650.6	526.6
SM 36:2	767.552	735.605	767.5464	735.5987	-7.4	-8.8		Yes			676.6	552.6
SM 40:1	825.622	793.677	825.6246	793.6769	2.7	-0.4		Yes			734.7	610.7
SM 42:2	851.638	819.693	851.6403	819.6926	2.5	-0.2		Yes			760.7	636.7

Table S5. **cntd.**

									Diagnostic fragment ions (Li+ precursor)	
Lipid species	*m/z* (K+)	*m/z* (Li+)	Exact mass (K+)	Exact mass (Li+)	ppm (K+)	ppm (Li+)	Major FA composition	Confirmed by MS/MS	DAG (H+)	DAG (Li+)
TG 50:2	869.697	ND	869.6995	837.7518	2.6					
TG 52:2	897.729	865.780	897.7308	865.7831	2.1	3.3	16:0/18:1/18:1	Yes	577.5, 603.5	583.5, 609.5
TG 52:3	895.713	863.765	895.7151	863.7674	2.6	3.2	16:0/18:1/18:2	Yes	577.5, 575.5, 601.5	583.5, 581.5, 607.5
TG 52:4	893.697	861.750	893.6995	861.7518	3.4	2.4				
TG 54:2	925.764	ND	925.7621	893.8144	-1.5					
TG 54:3	923.744	891.797	923.7464	891.7987	2.3	2.5	18:1/18:1/18:1	Yes	603.5	609.5
TG 54:4	921.728	889.781	921.7308	889.7831	2.7	2.6	18:1/18:1/18:2	Yes	601.5, 603.5	607.5, 609.5
TG 54:5	919.712	887.766	919.7151	887.7674	3.9	1.7				
TG 54:6	917.696	ND	917.6995	885.7518	4.4					

Table S6. Commonly observed measurement induced fragments

Lipid	Intact species (K+)	Loss of $N(Me)_3$
PC 32:0	772.523	713.449
PC 32:1	770.507	711.433
PC 34:1	798.538	739.464
PC 34:2	796.522	737.448
PC 34:3	794.508	735.434
PC 36:1	826.574	767.500
PC 36:2	824.553	765.479
PC 36:3	822.538	763.464
PC 36:4	820.522	761.448
PC 38:4	848.553	789.479
PC 38:6	844.522	785.448
SM 34:1	741.529	682.455
SM 40:1	825.622	766.548
SM 42:2	851.638	792.564

Supplementary References

1. Jha P, Knopf A, Koefeler H, Mueller M, Lackner C, Hoefler G, Claudel T, et al. Role of adipose tissue in methionine–choline-deficient model of non-alcoholic steatohepatitis (NASH). Biochimica et Biophysica Acta (BBA) - Molecular Basis of Disease 2014;1842:959-970.

2. Wiklund S, Johansson E, Sjöström L, Mellerowicz EJ, Edlund U, Shockcor JP, Gottfries J, et al. Visualization of GC/TOF-MS-Based Metabolomics Data for Identification of Biochemically Interesting Compounds Using OPLS Class Models. Analytical Chemistry 2008;80:115-122.

3. Yew Tan C, Virtue S, Murfitt S, Roberts LD, Phua YH, Dale M, Griffin JL, et al. Adipose tissue fatty acid chain length and mono-unsaturation increases with obesity and insulin resistance. Scientific Reports 2015;5:18366.
